# Ticks and tick-borne diseases on southwestern Indian Ocean islands

**DOI:** 10.1186/s13071-026-07570-3

**Published:** 2026-07-18

**Authors:** Antsa Rakotonirina, Nil Rahola, Dmitry A. Apanaskevich, Miora Felaniaina Andrianandrasana, Thierry Baldet, Mihary Reine Razafimamonjy, Luciano Michaël Tantely, Mandaniaina Radotiana Andriamiarimanana, Malala Nirina Rakotomanga, Steven M. Goodman, Marlène Dupraz, Diego Ayala

**Affiliations:** 1 Medical Entomology Unit, Institut Pasteur de Madagascar, 101 Antananarivo, Madagascar; 2 UMR MIVEGEC, CNRS, IRD, University of Montpellier, Montpellier, France; 3 US National Tick Collection, The James H. Oliver, Jr. Institute for Coastal Plain Science, Georgia Southern University, Statesboro, GA 30460 USA; 4 Department of Biology, Georgia Southern University, Statesboro, GA 30460 USA; 5 UMR ASTRE, CIRAD, Plateforme Technologique CYROI, Sainte Clotilde 97491, La Réunion, France; 6 UMR ASTRE, Univ de Montpellier, CIRAD, INRAE, Montpellier, France; 7 Association Vahatra, 101 Antananarivo, Madagascar; 8 Field Museum of Natural History, Chicago, IL 60605 USA

**Keywords:** Ticks, Tick-borne diseases, One Health, Southwestern Indian Ocean islands

## Abstract

**Background:**

Tick-borne diseases represent a growing global health concern, affecting both humans and animals, with an increasing number of disease outbreaks reported worldwide. On the southwestern Indian Ocean islands (SWIO)—including the Comoros, Madagascar, Mauritius, La Réunion, Rodrigues and the Seychelles—the diversity of tick species and the pathogens they transmit remain poorly understood, posing a considerable impediment to improving public and animal health.

**Methods:**

Here, we present the first comprehensive summary of tick species diversity and tick-borne pathogens in the SWIO region.

**Results:**

In this review, we list 42 tick species across seven genera with *Haemaphysalis* (13 species) and *Ixodes* (13 species) being the most species-rich. Although many species appear endemic to the SWIO region and, in numerous cases, to specific islands, the main vectors involved in pathogen transmission are cosmopolitan and introduced taxa. Tick-borne diseases such as babesiosis, borreliosis, and cowdriosis circulate on different islands in the SWIO region, particularly affecting cattle and small ruminants. Importantly, we highlight critical issues related to taxonomic identifications, molecular diagnostics, vector competence studies, and the integration of One Health approaches (i.e., an integrated and systemic approach that aims to sustainably balance and optimize the health of humans, animals, and ecosystems), which are further accentuated by the socio-economic and ecological contexts of each island.

**Conclusions:**

Addressing these gaps will be essential to strengthen surveillance, assess risks, and design effective preventive and control strategies. We conclude that the insular context creates favorable conditions for the implementation of regional One Health strategies aimed at improving early detection, preparedness, and response to tick-borne diseases.

**Graphical Abstract:**

**Figure:**
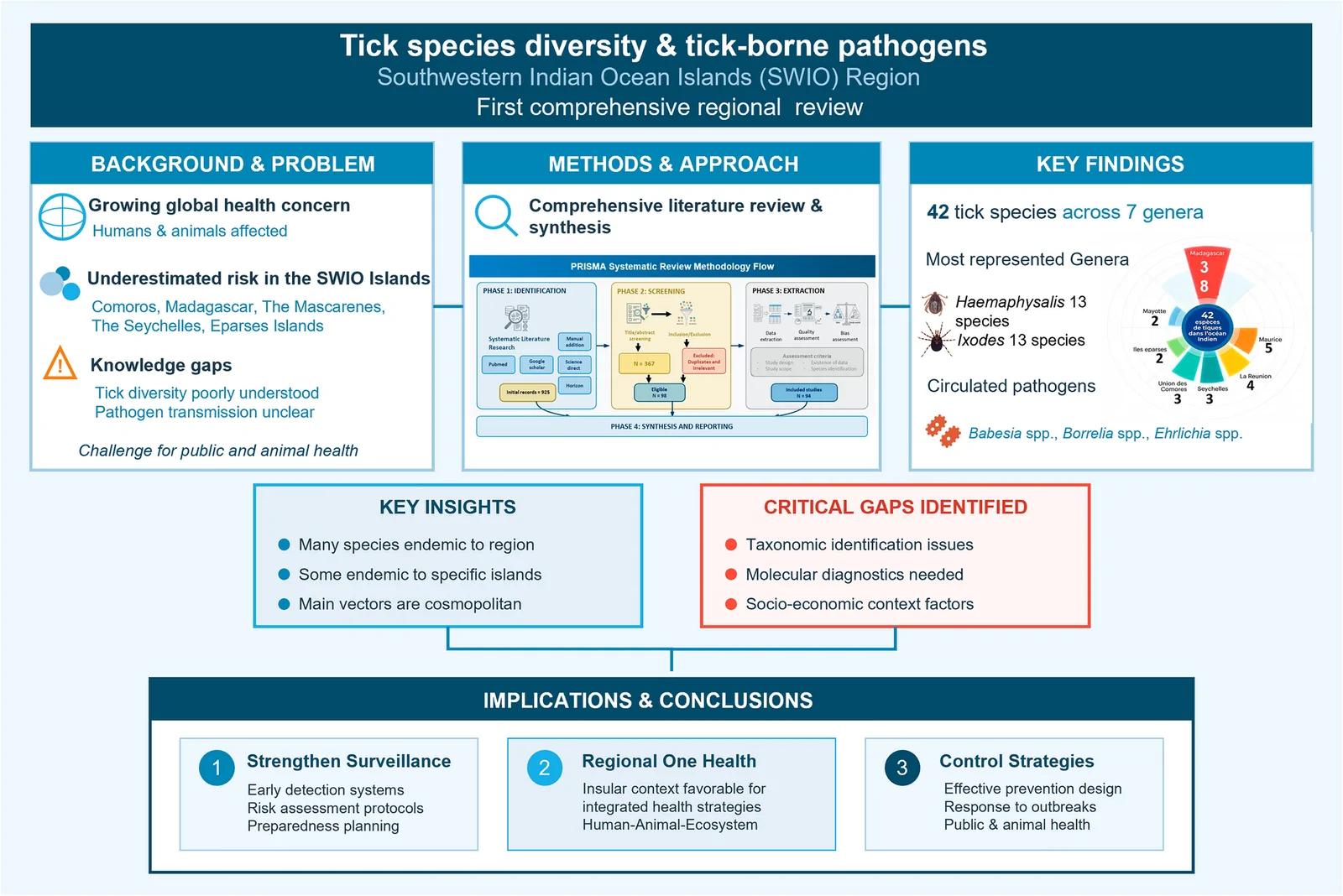

## Background

### General aspects on ticks and their impact on animal and public health

Ticks are obligate hematophagous ectoparasites of different animals [1] and found nearly worldwide, from tropical to more temperate regions [2] as well as in Arctic and Antarctic regions [3, 4]. Animals and ticks have long coexisted, with the earliest direct evidence of a tick–host association dating back to the Cretaceous, approximately 99 million years ago, involving ticks parasitizing feathered dinosaurs [5]. Since the domestication of animals around 11,500 years ago [6], humans have become increasingly exposed as new tick hosts [1]. Although only a few species are particularly well adapted to biting domestic animals and humans [7], many others can act as bridge vectors, transmitting pathogens between wildlife, domestic animals, and humans.

Ticks are indeed responsible for transmitting a broad range of pathogens among arthropod vectors to domestic animals [8] and some pathogens also affecting humans [1]. These include bacteria, parasites, and viruses, several of which cause serious health and economic impacts at the local or worldwide scale. For example, Lyme disease caused by spirochetes of the *Borrelia burgdorferi* sensu lato complex is the most common tick-borne disease (TBD) in the Northern Hemisphere, occurring throughout temperate regions of North America and Europe [9]. Other *Borrelia* species are responsible for tick-borne relapsing fevers across many tropical and subtropical regions, with case-fatality rates of up to 5% [10]. Numerous *Rickettsia* species cause spotted fever rickettsioses throughout the Americas, Asia, the Mediterranean basin, Africa, and tropical northern Australia [11]. Additional TBDs include babesiosis, bartonellosis, and various forms of ehrlichiosis and anaplasmosis, all of which occur globally. Q fever is widespread worldwide, while tularemia is reported in Europe, Japan, North America, and regions of the former USSR [11]. Viruses of the tick-borne encephalitis complex, endemic across much of Europe and Asia, can cause fatality rates of up to 15% after infection progresses to the neurological phase [12]. Other viral diseases such as the widespread Crimean–Congo hemorrhagic fever and the severe fever with thrombocytopenia syndrome, which circulates in eastern Asia, are associated with high mortality rates [11, 13, 14] further highlighting the public health significance of ticks. Beyond transmitting pathogens, ticks also directly affect animal behavior and well-being. Heavy infestations can cause significant blood loss leading to anemia, provoke severe allergic reactions to salivary toxins, and induce chronic stress and irritation [15]. They can also contribute to immunosuppression and considerable energy loss [16].

### The SWIO: biogeography and mammalian hosts of ticks

The southwestern Indian Ocean (SWIO) region comprises a mosaic of inhabited islands, including the Comoros, Madagascar, the Mascarenes (including Mauritius, La Réunion, and Rodrigues), and the Seychelles, governed by different nations, as well as uninhabited territories such as the Eparses Islands. These land masses differ greatly in terms of size and ecological conditions, ranging from large, near continental size to small coral atolls. Madagascar stands out from the others due to its considerable vertebrate diversity, spread across a range of different vegetation types, and its very high levels of endemism [17].

As mammals are important hosts of ticks, some details on the SWIO fauna are warranted. Madagascar has four extant mammal groups that are endemic to the island, each representing monophyletic lineages [18]: (1) The Primates or lemurs, composed of 110 species and five different families (Cheirogaleidae, Lepilemuridae, Lemuridae, Indriidae, and Daubentoniidae). Most species are strictly forest-dwelling. Some taxa have been introduced to the islands of the Comoros Archipelago. (2) Tenrecidae or tenrecs, classified in the order Afrosoricida and comprised of 31 species that are placed in three different subfamilies (Geogalinae, Oryzorictinae, and Tenrecinae). The majority of these species live in natural forest settings, with the exception of the Tenrecinae, which can also be found in open areas of natural or anthropogenic origin. The Tenrecinae have spines at least on their dorsum and are often infested with ticks, while the other two families have standard fur. (3) Nesomyinae or endemic rodents, which are part of the family Nesomyidae and made up of 28 species. These rodents for the most part occur in natural forests. (4) Eupleridae or endemic carnivorans and with seven currently recognized species placed in three different subfamilies (Cryptoproctinae, Euplerinae, and Galidinae), which live mainly in natural forests.

On other islands in the SWIO, there are no native non-flying mammals, and the following groups have been introduced and including on Madagascar: (1) Soricidae or shrews, part of the order Soricomorpha, and with two different species in the genus *Suncus*. These have been able to colonize natural forest settings and often occur as human commensals. (2) Muridae or introduced rodents, part of the order Rodentia, and with three species in two different genera (*Mus* and *Rattus*). These different rodents are generally human commensals, but can also be found in natural forest habitats. (3) Human commensals composed of Felidae or cats of the genus *Felis*, Canidae or dogs of the genus *Canis*, and a range of Artiodactyla including pigs, cows, deer, goats, and sheep. (4) Viverridae or the Indian civet of the genus *Viverricula*, which occurs in a feral state. Also, a spiny tenrec of the subfamily Tenrecinae, *Tenrec ecaudatus*, originally endemic to Madagascar, has been broadly introduced to other islands in the SWOI.

With regard to flying mammals, Madagascar hosts approximately 44 bat species belonging to eight families (Pteropodidae, Hipposideridae, Rhinonycteridae, Emballonuridae, Molossidae, Myzopodidae, Vespertilionidae, and Miniopteridae,), of which 35 species (80%) are endemic to the island [19]. Most Malagasy bat taxa are thought to have originated from mainland Africa [20]. On other islands in the SWIO, 14 bat species have been recorded [21].

### Knowledge gaps and emerging risks of ticks and TBDs in the SWIO region

The different landmasses in the SWIO region display marked differences in socio-economic development, which results in very diverse contexts for public and animal health surveillance systems. The regular movement of animals, particularly through the trade of cattle, goats, and other livestock between continental Africa and the SWIO, as well as informal exchanges and transport among the islands themselves, provides multiple pathways for the introduction and circulation of pathogens. Such exchanges amplify the risk of vector-borne disease emergence and spread across the region. Within this context, the SWIO constitutes a particularly sensitive environment for ticks and TBDs, where both endemic and introduced species can establish themselves and pose growing threats to human and animal populations.

Despite the ecological and biogeographical importance of the region, knowledge of ticks and TBDs in the SWIO remains dispersed in the literature. Available information is largely derived from isolated studies conducted on a limited number of islands. This unevenness of data largely reflects the lack of systematic surveillance, diagnostic capabilities, and sustained monitoring programs both within and between these island nations, as well as the logistical constraints inherent to island settings. Moreover, the data currently available has been largely influenced by investigations targeting pathogens of recognized veterinary and public health importance. Consequently, most documented tick-borne agents in the SWIO are largely those of domestic livestock, such as pathogens affecting cattle health and productivity or ruminant diseases [22–25] with significant economic impact. A small number of zoonotic agents have also been identified through studies focused on human exposure [26, 27]. In contrast, the role of wildlife in the maintenance and circulation of tick-borne pathogens remains poorly understood, particularly in biodiversity hotspots such as Madagascar, where high levels of mammalian endemism may favor unique tick–host–pathogen associations. On this island-nation, rodents, lemurs, and other wild mammals could serve as reservoirs for unknown agents that might spill over to livestock or humans, but this aspect remains largely unexplored.

To build an effective inter-island and inter-nation surveillance system in the SWIO, it is essential to first compile a comprehensive review of ticks and TBDs in the region. This foundational synthesis is critical for designing targeted monitoring and intervention strategies tailored to the ecological and socio-economic diversity of the islands. This highlights the urgent need for integrated research that includes domestic animals, wildlife, and humans to uncover both established and presumably undiscovered pathogens. Consequently, the present review seeks to consolidate and synthesize existing knowledge in the SWIO on tick species, their distribution, hosts, and the pathogens they transmit. By integrating available data, identifying key knowledge gaps, and highlighting priority areas for future research, we aim to provide a framework that will support the development of coordinated surveillance, risk assessment, and control strategies at the regional level.

## Methods

### Search Strategy

A systematic literature review was conducted to identify relevant publications on ticks and TBDs in the SWIO. The search strategy followed the Preferred Reporting Items for Systematic Reviews and Meta-Analyses (PRISMA) guidelines [28]. Search entries used in PubMed (https://pubmed.ncbi.nlm.nih.gov/), Horizon (https://horizon.documentation.ird.fr/exl-php/accueil), ScienceDirect (https://www.sciencedirect.com/), and Google Scholar (https://scholar.google.com/), available from 1954 to 2025 (search completed in February 2026). Search terms combined TBDs, tick-related keywords (ticks, Argasidae, Ixodidae, *Amblyomma*, *Argas*, *Haemaphysalis*, *Ixodes, Rhipicephalus*, etc.) with geographic identifiers (Indian Ocean region as well as the name of each SWIO landmass: Madagascar, Comoros, Seychelles, Mauritius, La Réunion, etc*.*). We also used the term Africa Region to include continental Africa and its associated islands, Malagasy Region as Madagascar and neighboring islands, and Afro-Malagasy Region as the combination of the previous mentioned two regions.

### Inclusion and exclusion criteria

Publications were included if they reported on ticks or TBDs within the SWIO, including aspects related to tick occurrence, distribution, biology, ecology, host associations, acaricide resistance, or pathogen detection in domestic animals, wildlife and/or humans. Peer-reviewed articles, chapters in books, theses, and internship reports were considered. Duplicate records retrieved from different databases and websites were identified and removed. Exclusion criteria also included studies conducted outside the SWIO, conference material without full article, or those unrelated to ticks and tick-borne diseases were excluded. Only publications in English or French were included.

### Screening and selection

We conducted a two-stage literature screening process. First, all retrieved records were screened at the title and abstract level to identify potentially relevant studies. Articles that met the initial criteria were then subjected to a full-text assessment to determine their eligibility based on predefined inclusion and exclusion criteria.

### Data extraction and analysis

The selection process was documented using a PRISMA flow diagram [29]. From each eligible study, we extracted information on tick species and life stage, geographic location and island, host species, acaricide resistance, diagnostic method (morphological or molecular), and pathogens detected. Data were compiled into summary tables and on a map to describe the diversity and distribution of ticks and tick-borne pathogens across the SWIO region. In this review, we define a species as a “potential vector” when (1) it has been found naturally infected with a given pathogen in the field, (2) when its abundance has been shown to correlate with pathogen prevalence, or (3) when it has been collected in high densities on an infected host. A species defined as a “confirmed vector” is when its ability to transmit the pathogen to a host has been demonstrated under controlled experimental conditions.

## Results

**Fig. 1:**
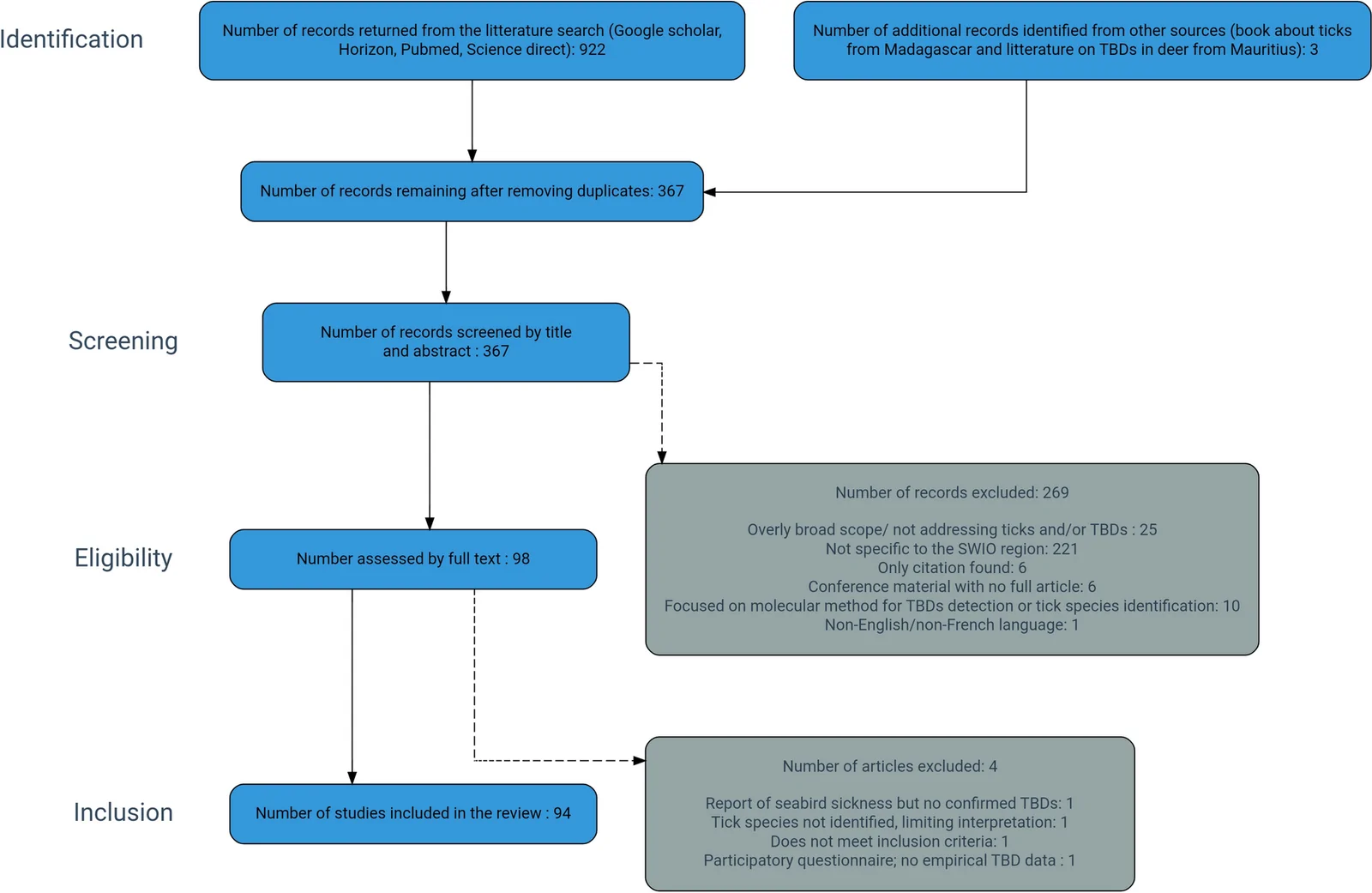
Flow diagram of search and selection of eligible publications

The initial search yielded 925 articles: 922 retrieved from open-source databases (including three chapters in edited volumes), two books on the tick fauna of Madagascar and their respective hosts, and one additional publication on TBDs in deer from Mauritius; the latter three were added manually. All records were imported into Mendeley, and 558 duplicates were removed. Of the remaining 367 article titles, abstracts, and subsequently full texts were screened, resulting in the exclusion of 273 studies that did not meet the inclusion criteria. Consequently, a total of 94 articles were included in this review (Fig. 1).

### Summary of findings

**Fig. 2:**
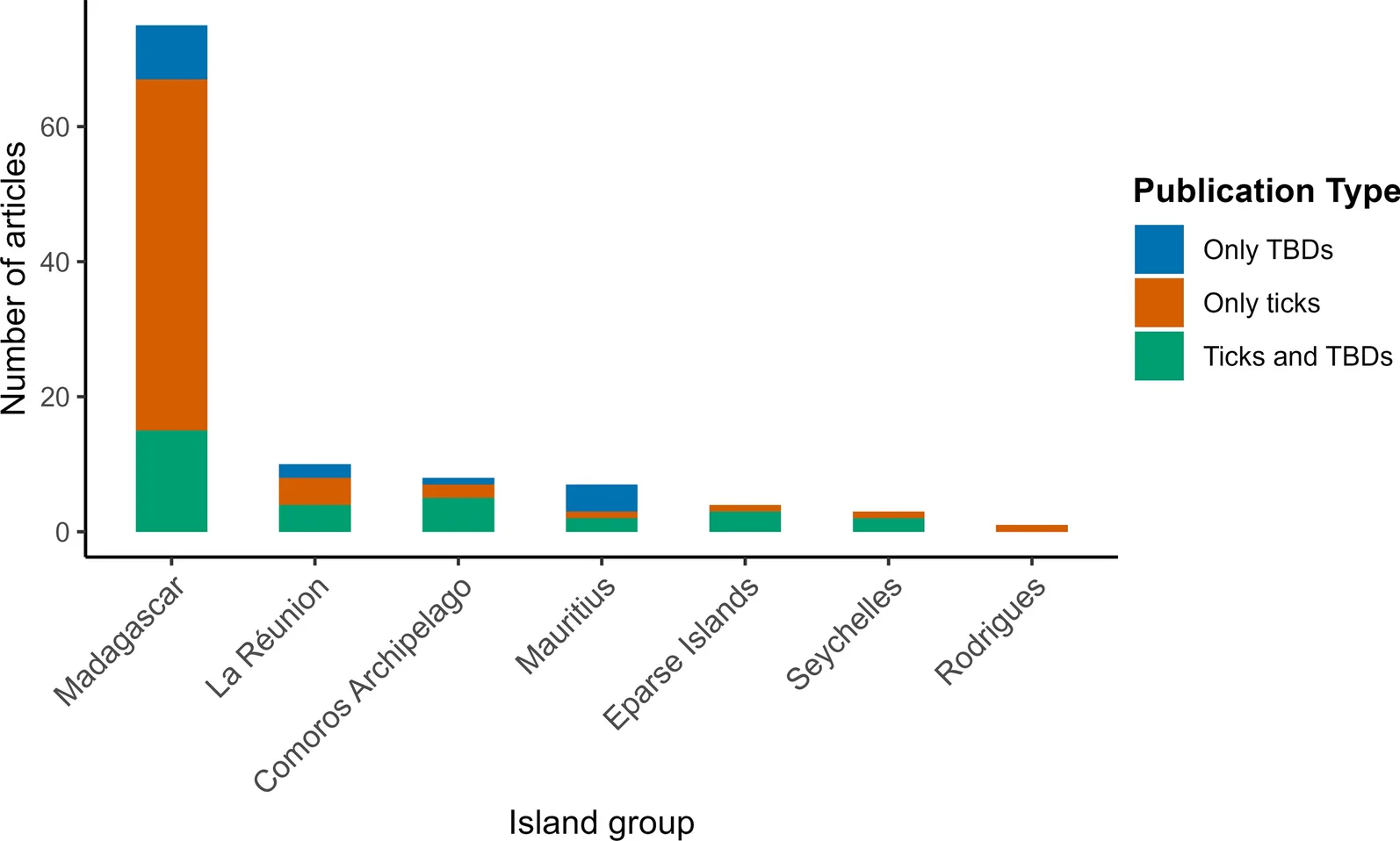
Country-level distribution of scientific publications focusing on ticks, tick-borne diseases (TBDs), or both in the southwestern Indian Ocean island region

Geographically, Madagascar accounted for the majority of publications on ticks and TBDs (Fig. 2) (*n* = 75), five of which were shared with other countries or archipelagos. This was followed by La Réunion (*n* = 10; five shared records), the Comoros Archipelago (*n* = 8; three shared records), Mauritius (*n* = 7; four shared records), the Seychelles (*n* = 3), the Eparses Islands (*n* = 4; three shared records), and Rodrigues (*n* = 1, shared record).

Overall, the included studies addressed a range of topics, notably the diversity and distribution of tick species, as well as aspects of their biology, taxonomy, ecology, and resistance to acaricides. Several studies also focused on the pathogens associated with ticks and their potential/confirmed role in pathogen transmission.

### Diversity and distribution of ticks on the southwestern Indian Ocean islands

**Fig. 3:**
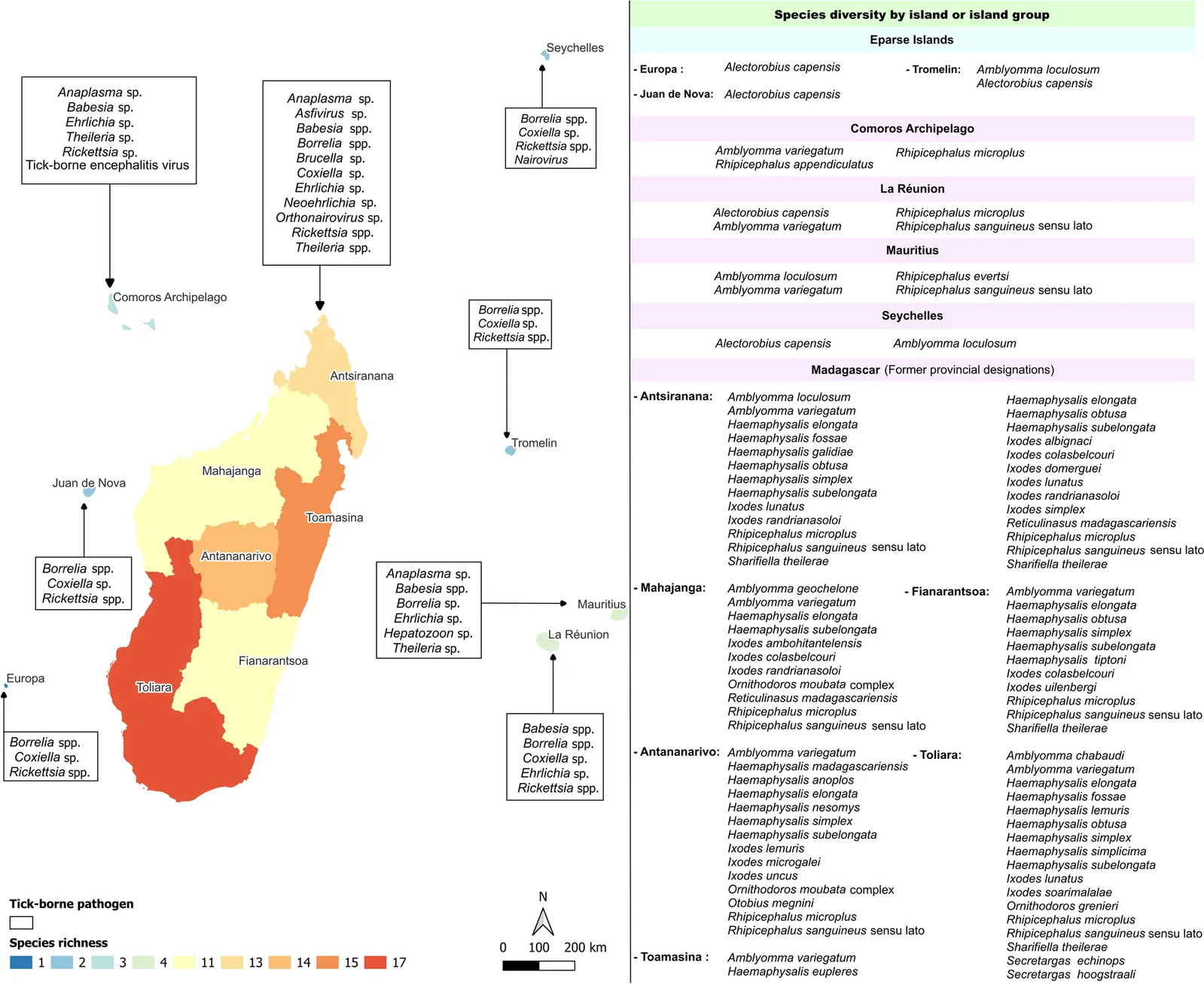
Spatial distribution of ticks and tick-borne pathogens across the southwestern Indian Ocean Island region. For Madagascar, data are presented using the former six-provincial administrative system, which has been replaced by the current regional division, to simplify the representation of tick distributions

There are 1033 tick species worldwide, with 800 belonging to the family Ixodidae, 221 to the family Argasidae, 11 species to the family Nuttalliellidae and one species to the family Khimairidae [1, 30, 31] Within the SWIO region, a total of 42 tick species have been documented to date, seven Argasidae and 35 Ixodidae (Table 1; Fig. 3).

**Table 1:** List of recognized tick species as of 1 January 2026 on southwestern Indian Ocean islands (SWIO) and their associated pathogens. Excluding domestic animals, species names preceded by an asterisk are introduced to Madagascar

Tick species	Described stages (sexes)	Vertebrate hosts	Associated pathogens in the SWIO	Distribution in the SWIO	Reference
*Alectorobius capensis *(Neumann, 1901)	L, N, A (M and F)	Seabird: *Onychoprion fuscatus*	*Rickettsia hoogstraalii, R. bellii, Borrelia* sp., Soldado virus	Bird, Europe, Juan de Nova, La Réunion, Tromelin	[3, 31, 34, 36, 38, 47]
*Ornithodoros grenieri * Klein, 1965	L, N, A (M and F)	Rodent: *Hypogeomys antimena*	No information	Madagascar	[32, 47]
*Ornithodoros porcinus* Walton, 1962	L, N, A (M and F)	Domestic pigs, Humans	African Swine Fever virus (*Asfivirus*)	Madagascar	[32, 43, 44]
*Otobius megnini* (Dugès, 1883)	L, N, A (M and F)	Cattle, Dogs, Horses, Sheep, Humans	*Coxiella burnetii*	Madagascar	[32, 47]
*Reticulinasus madagascariensis* (Hoogstraal, 1962)	L	Bats: Species not determined	No information	Madagascar	[31, 32, 47]
*Secretargas echinops* (Hoogstraal, Uilenberg & Blanc 1967)	L, N, A (M and F)	Tenrec: *Echinops telfairi, Geogale aurita*	*Rickettsia africae*	Madagascar	[31, 32, 46, 47]
*Secretargas hoogstraali* (Morel & Vassiliades, 1965)	L, N, A (M and F)	Reptiles: *Oplurus grandidieri, O. fierinensis, O. quadrimaculatus*	No information	Madagascar	[31, 32, 47]
*Amblyomma chabaudi* Rageau, 1964	L, N, A (M and F)	Tortoises: *Pyxis arachnoides, Astrochelys radiata*	*Rickettsia africae*	Madagascar	[31, 32, 46, 47, 49]
*Amblyomma geochelone* Durden, Keirans & Smith, 2002	A (M and F)	Tortoise: *Geochelone yniphora*	No information	Madagascar	[31, 47, 50]
*Amblyomma loculosum* Neumann, 1907	L, N, A (M and F)	Lizards, Marine birds, Humans	*Rickettsia africae, Coxiella* sp. Aride virus	Aride, Bird, Madagascar, La Réunion, Mauritius, Seychelles, Tromelin	[31, 34, 38, 47, 51–53]
*Amblyomma. variegatum *(Fabricius, 1794)	L, N, A (M and F)	Birds, Cattle, Dogs, Donkeys, Horses, Poultry, Sheep, Humans	*Anaplasma marginale, Borrelia* sp., *Coxiella burnetii, Ehrlichia ruminantium, Rickettsia africae, Theileria parva*	Comoros, Madagascar, La Réunion, Mauritius	[23, 26, 32, 59, 62–65, 67]
*Haemaphysalis anoplos* Hoogstraal, Uilenberg & Klein, 1967	A (F)	Rodent: *Eliurus webbi, Nesomys rufus*	No information	Madagascar	[31, 32, 47, 68]
*Haemaphysalis elongata* Neumann, 1897	L, N, A (M and F)	Tenrecs: *Setifer setosus, Tenrec ecaudatus, Hemicentetes nigripes, H. semispinosus, Echinops telfairi, Nesogale talazaci* Carnivora: *Galidictis fasciata* Bats Rodent: *Rattus rattus Humans	*Babesia brygooi*	Madagascar	[31, 32, 47, 70]
*Haemaphysalis eupleres* Hoogstraal, Kohls & Trapido, 1965	A (M and F)	Carnivora: *Eupleres goudotii* and *Galidia elegans*	No information	Madagascar	[31, 32, 47]
*Haemaphysalis fossae* Hoogstraal, 1953	A (M and F)	Carnivora: *Fossa fossa*, *Viverricula indica	*Babesia*	Madagascar	[31, 32, 47]
*Haemaphysalis galidiae* Apanaskevich & Goodman, 2020	A (M and F)	Carnivora: *Galidia elegans*	No information	Madagascar	[31, 47, 73]
*Haemaphysalis lemuris* Hoogtraal, 1953	N, A (M and F)	Lemurs: *Cheirogaleus crossleyi, Eulemur fulvus, Lemur catta, Lepilemur ruficaudatus, L. edwardsi, L. mustelinus, Microcebus griseorufus, M. rufus, Prolemur simus, Propithecus diadema, P. verreauxi, Varecia variegata*, Birds: *Schetba rufa*, *Cyanolanius madagascarinus*, Humans	*Borrelia* spp., *Candidatus*, *Neoehrlichia* sp., *Babesia* sp.	Madagascar	[31, 32, 47, 74, 75, 77, 81]
*Haemaphysalis madagascariensis* Colas-Belcour & Millot, 1948	A(M)	Birds: *Centropus toulou, Artamella viridis, Coua reynaudii*	No information	Madagascar	[31, 32, 47]
*Haemaphysalis nesomys* Hoogstraal, Uilenberg & Klein, 1966	A(M)	Rodents: *Nesomys rufus*	No information	Madagascar	[31, 32, 47]
*Haemaphysalis obtusa *Dönitz, 1910	L, N, A (M and F)	Carnivora: *Canis lupus, Fossa fossana, Galidia elegans, Cryptoprocta ferox, Galidictis fasciata, Eupleres goudotii, *Viverricula indica, dogs, Tenrec: *Nesogale talazaci*, *Hemicentetes semispinosus*, Rodents: *Nesomys rufus*, *Rattus rattus)	*Babesia* sp.	Madagascar	[31, 32, 47]
*Haemaphysalis simplex* Neumann, 1897	L, N, A (M and F)	Tenrecs: *Setifer setosus, Echinops telfairi* Lemurs: *Microcebus murinus, M. griseorufus*, *Propithecus verreauxi*, Bird: *Lophotibis cristata*, Tenrecs: *Echinops telfairi, Tenrec ecaudatus, Nesogale talazaci, Setifer setosus*, Rodents: *Nesomys rufus,* *Rattus rattus	*Babesia brygooi, Borrelia duttoni*, *Rickettsia africae*	Madagascar	[31, 32, 46, 47, 74, 75]
*Haemaphysalis simplicima* Hoogstraal & Wassef, 1979	N, A (M and F)	Tenrecs: *Echinops telfairi*, *Geogale aurita, Setifer setosus*, Bats: *Macronycteris commersoni*	No information	Madagascar	[31, 32, 47]
*Haemaphysalis subelongata * Hoogstraal, 1953	L, N, A (M and F)	Tenrecs: *Echinops telfairi, Hemicentetes semispinosus, Tenrec ecaudatus*, Rodents: *Rattus rattus, *Macrotarsomys bastardi*	No information	Madagascar	[31, 32, 47]
*Haemaphysalis tiptoni* Hoogstraal, 1953	A (M and F)	Tenrecs: *Hemicentetes nigriceps*, *H. semispinosus, Setifer setosus, Tenrec ecaudatus*	No information	Madagascar	[31, 32, 47]
*Ixodes albignaci* Uilenberg & Hoogstraal, 1969	L, N, A (M and F)	Tenrecs: *Microgale cowani, M. drouhardi, M. gracilis, M. monticola, M. taiva, Nesogale talazaci*, *Oryzorictes hova, O. talpoides* *Suncus murinus, Rodents: *Eliurus majori, E. tanala, Nesomys rufus,* *Rattus rattus	No information	Madagascar	[31, 32, 47, 72]
*Ixodes ambohitantelensis* Englert, Goodman & Apanaskevich, 2023	A (F)	Tenrec: *Microgale majori*, Rodent: *Mus musculus	No information	Madagascar	[31, 47, 85]
*Ixodes colasbelcouri* Arthur, 1957	L, N, A (M and F)	Tenrecs: *Microgale cowani*, *Nesogale dobsoni, N. talazaci, Tenrec ecaudatus*, Rodents: *Brachytarsomys albicauda, Brachyuromys betsileoensis*, *Eliurus tanala, Gymnuromys roberti, Nesomys rufus,* *Rattus rattus	*Rickettsia felis*	Madagascar	[31, 32, 47]
*Ixodes domerguei *Uilenberg & Hoogstraal, 1965	A (F)	Birds: *Newtonia brunneicauda, Nelicurvius nelicourvi*	No information	Madagascar	[31, 32, 47]
*Ixodes lemuris* Arthur, 1958	L, N, A (M and F)	Lemur: *Cheirogaleus crossleyi, C. sibreei, Eulemur fulvus, E. rufus, Indri indri, Lepilemur mustelinus, Microcebus rufus, Propithecus diadema*, Rodent: *Rattus rattus	No information	Madagascar	[31, 32, 47]
*Ixodes lunatus* Neumann, 1907	A (F)	Tenrecs: *Hemicentetes semispinous, Microgale cowani, Setifer setosus, Tenrec ecaudatus*, Rodent: *Nesomys audeberti*	No information	Madagascar	[31, 32, 47]
*Ixodes microgalei* Apanaskevich, Soarimalala & Goodman, 2013	A (F)	Tenrecs: *Microgale parvula*, *M. soricoides, Nesogale dobsoni*	No information	Madagascar	[31, 47, 91]
*Ixodes nesomys* Uilenberg & Hoogstraal, 1969	A (F)	Rodent: *Nesomys rufus*	No information	Madagascar	[31, 32, 47]
*Ixode randrianasoloi *Uilenberg & Hoogstraal, 1969	A (F)	Tenrec: *Nesogale talazaci* Rodent: *Rattus rattus	No information	Madagascar	[31, 32, 47]
*Ixodes simplex* Neumann, 1906	L, N, A (M and F)	Bats: *Minopterus egeri, M. majori, M. manavi, M. sororculus, Myotis goudoti*	No information	Madagascar	[31, 47]
*Ixodes soarimalalae* Apanaskevich & Goodman, 2020	A (F)	Tenrec: *Microgale cowani*	No information	Madagascar	[92]
*Ixode uilenbergi* Apanaskevich & Goodman, 2020	A (F)	Tenrecs: *Hemicentetes nigriceps, Nesogale dobsoni, Oryzorictes hova*	No information	Madagascar	[31, 47, 92]
*Ixode uncus* Apanaskevich & Goodman, 2020	A (F)	Tenrecs: *Microgale soricoides, Nesogale dobsoni,*	No information	Madagascar	[31, 47, 92]
*Rhipicephalus appendiculatus* Neumann, 1901	L, N, A (M and F)	Cattle	*Ehrlichia ruminantium, Rickettsia africae, Theileria parva*	Comoros, Mauritius	[56, 58, 59, 64]
*Rhipicephalus evertsi *Neumann, 1897	L, N, A (M and F)	Introduced rodent, Horses	*Babesia ovis, Theileria ovis*	Mauritius	[56]
*Rhipicephalus microplus* (Canestrini, 1888)	L, N, A (M and F)	Cattle, Deer, Dogs, Goat, Horses, Rodents, Sheep, Humans	*Anaplasma marginale, Babesia bovis, B. bigemina, Borrelia* sp. (probably *B. theileri*), *Coxiella burnetii, Ehrlichia ruminantium, Rickettsia africae,* Crimean Congo hemorrhagic fever virus	Comoros, Madagascar, Mauritius, La Réunion	[23, 46, 47, 57, 58, 64, 67]
*Rhipicephalus sanguineus* sensu lato (Latreille, 1806)	L, N, A (M and F)	Dogs	*Babesia canis*	La Réunion, Madagascar, Mauritius	[31, 32, 47, 56, 106]
*Sharifiella theilerae *(Hoogstraal, 1953)	L, N, A (M and F)	Tenrecs: *Setifer setosus*, *Tenrec ecaudatus*	No information	Madagascar	[31, 32, 47, 108]

*A* adult, *F* female, *L* larvae, *M* male, *N* nymph

An asterisk (*) denotes species introduced to Madagascar, excluding domestic animals

### Family Argasidae

This family is characterized by three key features: (1) the absence of a scutum, (2) the anterior placement of stigmata relative to the fourth coxae, and (3) the ventrally positioned capitulum [32]. We follow the taxonomic delineations of soft ticks proposed by Dantas-Torres et Mans (2026) [31]. In the SWIO region, published references indicate that species belonging to this family are often host-specific, sometimes parasitizing vertebrates at risk of extinction, particularly due to habitat loss and deforestation on Madagascar. Consequently, some of these tick species may also be threatened. Among the seven Argasidae species reported in the SWIO, four are potential vectors of bacterial and/or viral pathogens, and one is a confirmed vector of a virus (Table 1). The different species of this family present in the SWIO are described below in alphabetical order by genus and species.

#### Genus *Alectorobius* Pocock, 1907

This genus is distributed across different regions of the world and includes species that parasitize mammals (mainly bats) and birds. At present, 64 *Alectorobius* species have been described [31], one of which is found in the SWIO region.

#### *Alectorobius capensis* (Neumann, 1901)

Formerly known as *Carios capensis* [33], this species has been recorded from several islands in the SWIO, including La Réunion, the Seychelles (Bird Island), and the Eparses Islands (Europa, Juan de Nova, and Tromelin) [34, 35]. It parasitizes seabirds, specifically a species of tern (family Sternidae) [34, 36] and can also accidentally bite humans [37]. *Alectorobius capensis* on La Réunion, the Seychelles, and the Eparses Islands is a potential vector of different bacteria: *Borrelia* spp. (reported at low abundance) and *Rickettsia* spp. (probably *R. hoogstraalii* and *R. bellii*) [34, 38]. The tick population from Bird Island is a confirmed vector of Soldado virus [36].

#### Genus *Ornithodoros* Koch, 1837

The genus has a worldwide distribution and a diverse host range, including reptiles, birds, and mammal [40]. At present, *Ornithodoros* genus contains 47 species [31], of which two have been identified in the SWIO region.

#### *Ornithodoros grenieri* Klein, 1965

Endemic to Madagascar, it has been recorded in the central western portion of the island, where it parasitizes the Malagasy giant rat, *Hypogeomys antimena* of the endemic subfamily Nesomyinae [32]. Its role as a vector of pathogens remains unknown.

#### *Ornithodoros moubata* complex (Murray, 1877)

Several eyeless *Ornithodoros* species, collectively referred to as the *Ornithodoros moubata* complex, have been identified. On Madagascar, these ticks were initially described as *O. moubata porcinus* Walton, 1962 and were found along the western coast and in the Central Highlands, where they parasitize domestic pigs [32, 41]. Subsequent morphological comparisons by Uilenberg (1963), based on Walton’s description (1962), led to the conclusion that specimens collected from pig pens on Madagascar belonged to *O. porcinus* [42]. Subsequent molecular analysis suggested that the populations of *O. porcinus* from the Central Highlands are closely related to *O. porcinus domesticus* Walton, 1962 [43]. While this latter form is a potential vector of African Swine Fever virus on the island [43], to date, the presence of these species elsewhere in the SWIO has not been reported.

#### Genus *Reticulinasus* Schulze, 1941

This genus is distributed across different regions of the world and includes species that parasitize mainly bats. To date, 12 *Reticulinasus* species are recorded worldwide [31], one of which occurs in the SWIO region.

#### *Reticulinasus madagascariensis* (Hoogstraal, 1962)

Formerly known as *Ornithodoros madagascariensis,* this is an endemic species to Madagascar. The type material was collected along the road between Antananarivo and Mahajanga; however, no precise locality was recorded. Although it is known to parasitize bats (Chiroptera), much remains to be elucidated regarding its biology and role as a vector of pathogens [32]. After its description by Hoogstraal (1962) [39] in the genus *Ornithodoros*, a redescription of the species has been provided by Sonenshine [44].

#### Genus *Otobius* Banks, 1912

*Otobius* ticks occur in North America, Africa, and Asia, with their presence in the latter two continents resulting from accidental introduction and can bite a wide range of hosts. Two *Otobius* species are currently described [31], one of which occurs in the SWIO region.

#### *Otobius megnini* (Dugès, 1883)

This cosmopolitan tick is known from different countries in Africa, America, Asia, and Europe. In the SWIO region, it has been only recorded on Madagascar where it parasitizes a wide range of domestic animals, including horses, cattle, sheep, and dogs, and can bite humans [32]. This species occurs primarily in the Central Highlands, although, there appear to be no climatic barriers preventing its spread across most of the island. *Otobius megnini* has been implicated in creating conditions conducive to myiasis (as severe ear damage, inflammation, and necrotic tissue can lead to secondary maggot infections) and is a potential vector of the Q fever agent, *Coxiella burnetii* [32].

#### Genus *Secretargas* Hoogstraal, 1957

This genus currently comprises three described species [31], two of which are endemic to the SWIO region, whereas the third is widely distributed, occurring mainly in the Mediterranean region of North Africa and southwestern Europe, extending eastward to Afghanistan with only sporadic northern records, and also present in southern Africa (South Africa and Namibia) [32, 45]. Species within the genus can parasite lizards, micromammals, and rarely humans [32, 45].

#### *Secretargas echinops* (Hoogstraal, Uilenberg & Blanc, 1967)

Endemic to Madagascar and specifically in the southwest of the island, it parasitizes the spiny tenrec, *Echinops telfairi* [32, 46] and might also parasitize the large-eared tenrec *Geogale aurita* [47]. Laboratory attempts to introduce nymphs and adults of *S. echinops* on chickens, their natural host (*E. telfairi*), and humans were unsuccessful. Although partial engorgement was occasionally observed on rabbit ears, the nymphs failed to molt, and no oviposition occurred in adult females. This tick species is a potential vector of *Rickettsia africae,* the causative agent of African tick-bite fever [48].

#### *Secretargas hoogstraali* (Morel & Vassiliades, 1965)

Endemic to Madagascar, this species was collected in the southern regions of the island, where it parasitizes reptiles [32], specifically members of the genus *Oplurus* (family Opluridae) [47]. Despite several attempts to rear this tick species under laboratory settings, *S. hoogstraali* has proven reluctant to bite various hosts, including humans, rabbits, guinea pigs, chickens, and even reptiles. Its role as a vector of pathogens is unknown.

### Family Ixodidae

This family is characterized by three diagnostic features: (1) the presence of a scutum, (2) the posterior placement of stigmata relative to the fourth coxae, and (3) the anterior position of the capitulum [32]. In the SWIO region, species within this family parasitize a wide diversity of animals, some being host generalists and others host specific. Known hosts range from small endemic and introduced mammals to large domestic ruminants, with some species occasionally feeding on humans. Of the 35 Ixodidae species described in the SWIO, 13 are known or suspected vectors of bacteria, parasites, or viruses. The Ixodidae species present in the SWIO are listed below in alphabetical order by genus and species.

#### Genus *Amblyomma* Koch, 1844

Its distribution is worldwide, occurring mainly in humid tropical and subtropical regions, and nearly all terrestrial vertebrates serve as hosts. The genus comprises 139 species [31], four of which are found in the SWIO region.

#### *Amblyomma chabaudi* Rageau, 1964

Endemic to Madagascar, it was recorded in the south parasitizing endemic tortoises (family Testudinidae), including *Astrochelys radiata* and *Pyxis arachnoides* [32, 46, 49] as well as the endemic nesomyine rodent *Macrotarsomys bastardi* [46]. Studies have shown that the prevalence of *A. chabaudi* on tortoises is higher in disturbed habitats than in intact environments [49]. It is a potential vector of the agent of African tick-bite fever *Rickettsia africae,* according to molecular diagnostics, and may pose a potential health risk to human [48, 49].

#### *Amblyomma geochelone* Durden, Keirans & Smith, 2002

Endemic to Madagascar, this species has been recently described from the northwestern part of the island, where it parasitizes the Critically Endangered endemic tortoise *Astrochelys yniphora* (family Testudinidae) [50]. While the immature stages may feed on other vertebrates, adults are presumed to feed exclusively on this tortoise species. Its role as a potential vector of pathogen has not been studied.

#### *Amblyomma loculosum* Neumann, 1907

This species is found on Mauritius, Madagascar, and in the Seychelles (African Banks, Amirantes, Bird, Cousin, Goelette, Farquhar, Mahé, and Mamelles islands) and the Eparses Island (Tromelin), where adults and immatures parasitize a variety of seabirds (*Anous stolidus*, *Onychoprion fuscatus*, *Phaethon lepturus*, *Sterna dougallii*, *S. fuscata*, *Sula dactylatra*, and *S. leucogaster*), lizards (*Trachylepis wrightii*), and also humans [34, 35, 38, 51–53]. Its presence on multiple islands is likely facilitated by seabird migration. This tick demonstrates great adaptability to environmental conditions. Under laboratory conditions, its life cycle can be completed in just five months without entering diapause [52]. In contrast, field observations indicate a diapause period lasting 6–7 months, which synchronizes with the life cycle of its principal host, the sooty tern (*Onychoprion fuscatus*) [54]. Populations of *A. loculosum* from several landmasses in the SWIO region (including Aride, Bird, La Réunion, and Tromelin), have been found to be potential vectors of *Rickettsia africae* [34] and *Coxiella* sp. [38]. In the Seychelles, this tick has also been reported to be a potential vector of the Aride virus, which caused illness and death in laboratory mice following experimental infection [52].

#### *Amblyomma variegatum* (Fabricius, 1794)

Widely distributed across the African region [55], this tick is known to parasitize a diverse range of hosts, including birds, cattle, dogs, deer, donkeys, horses, poultry, sheep, and occasionally humans [32, 47, 56]. In the SWIO region, this species has been reported from the Comoros Archipelago (Anjouan, Grande Comores, Mohéli, and Mayotte), Madagascar, La Réunion, and Mauritius [57–59]. Although *A. variegatum* from Madagascar, where it has a broad distribution [60], shows a high adaptability to local temperature conditions [61] and survives both cold and dry seasons, laboratory experiments have shown that prolonged exposure to low temperatures is fatal [62]. In addition, cold temperatures extend the period for nymphs to molt [62]. One study suggests potential resistance to arsenic in *A. variegatum* [63]. In the SWIO region, this species is considered as the main tick vector of pathogens affecting ruminants. Populations from the Comoros Archipelago (Grande Comores, Anjouan, and Mohéli), Madagascar, and La Réunion are potential vectors of *Rickettsia africae* [26, 64, 65]. In addition, *A. variegatum* from Madagascar is a potential vector of *Anaplasma marginale*, *Borrelia* sp., and *Coxiella burnetii* [23, 48, 66, 67]. In the Comoros, the abundance of this species was found to be positively correlated with the prevalence of *Theileria parva* and *Ehrlichia ruminantium* antibodies in cattle, goat, and sheep [59].

#### Genus *Haemaphysalis* Koch, 1844

This genus has a worldwide distribution, parasitizing birds and mammals. It contains 173 species [31], 13 of which occur the SWIO region.

#### *Haemaphysalis anoplos* Hoogstraal, Uilenberg & Klein, 1967

Endemic to Madagascar, this species has been collected in the Central Highlands where it parasitizes the endemic rodents *Eliurus webbi* and *Nesomys rufus* [32, 47, 68]. Currently, no information is available about the tick’s biology and ecology. Its role as a pathogen vector is also unknown.

#### *Haemaphysalis elongata* Neumann, 1897

Endemic to Madagascar*,* this taxon is known to parasitize a range of hosts, including a variety of spiny and at least one furred Tenrecidae, endemic carnivorans, bats, introduced rodents, and occasionally humans [32, 47, 69, 70]. Its broad geographic distribution across the island probably reflects the fairly extensive distribution of its hosts. It is a potential vector of *Babesia brygooi* [32].

#### *Haemaphysalis eupleres* Hoogstraal, Kohls & Trapido, 1965

Endemic to Madagascar, this tick has been collected in the northeast of the island and the Central Highlands, where it is known to parasitize the endemic carnivorans, *Eupleres goudotii* and *Galidia elegans* [32, 47, 71, 72]. There is currently a lack of information regarding this tick’s biology, ecology, and possible role as a vector of pathogens.

#### *Haemaphysalis fossae* Hoogstraal, 1953

Endemic to Madagascar, it has been collected from the southeastern and the northeastern regions, and is known to parasitize mammals including the endemic carnivoran, *Fossa fossana*, and the introduced carnivoran, *Viverricula indica* [32, 47]. It is a potential vector of *Babesia* in *Cryptoprocta ferox* and *V. indica* [32].

#### *Haemaphysalis galidiae* Apanaskevich & Goodman, 2020

Endemic to Madagascar, it has been collected in the northern part of the island at elevations ranging from 400 to 900 m above sea level [73]. This species is known to parasitize *Galidia elegans,* an endemic carnivoran. Its role in the transmission of pathogens is currently unknown.

#### *Haemaphysalis lemuris* Hoogstraal, 1953

Endemic to Madagascar, it has been recorded in the southern and western regions, parasitizing both nocturnal and diurnal lemurs: *Avahi laniger*, *Cheirogaleus crossleyi*, *Eulemur fulvus*, *Lemur catta*, *Lepilemur* spp., *Microcebus* spp., *Prolemur simus*, *Propithecus* spp., and *Varecia variegata*; it is known to bite humans [47, 74–80]. This tick species has also been reported on birds, including those of the family Vangidae endemic to Madagascar [32]. Although understanding of its seasonal dynamics is limited, studies indicate a peak abundance of the nymphal stage on *Microcebus griseorufus* in October [74] and on *M. murinus* in August [75]. Interestingly, this ectoparasite preferentially infests adult male and subadult male hosts, with a significantly lower prevalence observed on adult females [77]. The microbiome of this tick has been found to be highly diverse, containing bacteria that are pathogenic or potentially pathogenic to lemurs [81]. It is a potential vector of pathogens belonging to the following genera: *Borrelia*, *Candidatus*, *Neoehrlichia*, and *Babesia* [32, 81].

A taxonomic entity (*Haemaphysalis* sp. « microcebi») with a questionable taxonomic status close to *H. lemuris* has recently been defined and appears to be endemic to Madagascar, specifically the northern and western parts of the island [82]. Although this tick can parasitize introduced *Rattus rattus*, it seems to prefer members of the endemic lemur genus *Microcebus,* and infestations of this tick’s nymphs peak in August [82, 83] and appear to be common in fragmented forests [82].

#### *Haemaphysalis madagascariensis* Colas-Belcour & Millot, 1948

Endemic to Madagascar, the exact collection locality of the type material of this species remains uncertain, although it is believed to come from between Antananarivo in the Central Highlands and Toamasina along the east coast. This species is known to parasitize *Centropus toulou*, an endemic bird of Malagasy Region [32] and other endemic birds: *Artamella viridis* and *Coua reynaudii* [47]. Its role as a vector of pathogens is unknown.

#### *Haemaphysalis nesomys* Hoogstraal, Uilenberg & Klein, 1966

Endemic to Madagascar, this species has been found across the Central Highlands [32] and parasitizes endemic rodents of the genus *Nesomys*. There is no information available regarding this tick’s biology, ecology, and role as a pathogen vector.

#### *Haemaphysalis obtusa* Dönitz, 1910

Endemic to Madagascar, this species has a wide distribution across the island, being found in the southeast, northeast, west, and the Central Highland regions [32, 72]. It parasitizes a number of endemic carnivores, as well as introduced carnivores including domestic dogs [32, 47]. The immature stages appear to parasitize certain spiny or furred species of Tenrecidae, as well as endemic Nesomyinae and introduced Muridae rodents. This tick is a potential vector of *Babesia* sp. [32].

#### *Haemaphysalis simplex* Neumann, 1897

Endemic to Madagascar, this species is distributed across the island, where it parasitizes a range of hosts, including spiny and furred tenrecs, lemurs, and introduced rats, as well as birds [32, 46, 75]. Studies have identified the nymphal stage of *H. simplex* on *Microcebus* mouse lemurs during the early part of the dry season (May–July) [74]. This tick species is a potential vector of *Babesia brygooi*, *Borrelia duttoni*, and *Rickettsia africae* [32, 48].

#### *Haemaphysalis simplicima* Hoogstraal & Wassef, 1979

Endemic to Madagascar, this species has been collected from the southern and southwestern regions. It is known to parasitize endemic spiny tenrec species [32] and bats [47]. Currently, there is no available information regarding its role as a pathogen vector.

#### *Haemaphysalis subelongata* Hoogstraal, 1953

Endemic to Madagascar, this tick has a widespread distribution on the island, including the east, north, south, and the Central Highlands [32, 72]. It parasitizes spiny tenrecs and both endemic and introduced rodents [47]. There is no available information regarding its role as a pathogen vector.

#### *Haemaphysalis tiptoni* Hoogstraal, 1953

Endemic to Madagascar, this species is known to parasitize spiny tenrec species [32, 47]. It has been found exclusively in the Central Highlands, specifically around the former Antananarivo Province and between Antsirabe and Fianarantsoa [32, 72]. Its role as a vector of pathogens is currently unknown.

#### Genus *Ixodes* Latreille, 1795

The genus has a worldwide distribution, including Antarctica, and parasitizes a wide range of hosts, notably mammals and birds. It is the largest tick genus, comprising 291 species [31], 13 of which are found in the SWIO region.

#### *Ixodes albignaci* Uilenberg & Hoogstraal, 1969

Endemic to Madagascar, this species has been found in the north, as well as Central Highlands, where it parasitizes various species of furred tenrecs, as well as introduced rodents [32, 47, 72, 84]. Researchers have suggested the presence of considerable morphological variability in this species or that it is a paraphyletic species complex [32]. Its role as a vector of pathogens is unknown.

#### *Ixodes ambohitantelensis* Englert, Goodman & Apanaskevich, 2023

Endemic to Madagascar, this species has been recently described from the Central Highlands and is known to parasitize furred tenrecs (*Microgale*) and introduced rodents (*Mus*) [85]. Its capacity to act as a vector of pathogens remains unknown.

#### *Ixodes colasbelcouri* Arthur, 1957

Endemic to Madagascar, this species has been found in the Central Highlands and the north, parasitizing endemic and introduced rodents and different spiny and furred tenrec species [32, 86]. It is a potential vector of *Rickettsia* sp., including *R. felis* [86].

#### *Ixodes domerguei* Uilenberg & Hoogstraal, 1965

Endemic to Madagascar and collected in the Central Highlands and the east; it is known to parasitize endemic birds [32, 87]. To date, no additional investigations on its biology or ecology have been addressed and its role as a pathogen vector is unknown.

#### *Ixodes lemuris* Arthur, 1958

Endemic to Madagascar. This species has been recorded in the Central Highlands and along the eastern slopes of the island, where is parasitizes lemurs and introduced rats [32, 47, 88–90]. Its role as a pathogen vector is currently unknown.

#### *Ixodes lunatus* Neumann, 1907

Endemic to Madagascar, it has been collected in the north, southeast, southwest, and the Central Highlands [32, 72]. This species parasitizes spiny tenrecs and endemic rodents [32, 88]. To date, its role as a pathogen vector is not documented.

#### *Ixodes microgalei* Apanaskevich, Soarimalala & Goodman, 2013

Endemic to Madagascar, this species is currently only known from the Central Highlands [91]. However, given the absence of evident geographical barriers, it is likely to occur in eastern moist evergreen forests. This species parasitizes several species of furred tenrecs. Its potential role as a vector of pathogens remains unknown.

#### *Ixodes nesomys* Uilenberg & Hoogstraal, 1969

This endemic species of Madagascar has been found to parasitize members of the endemic rodent genus *Nesomys* in the north and northwest portions of the island, as well as the Central Highlands [32, 72]. Its potential role as a pathogen vector is unknown.

#### *Ixodes randrianasoloi* Uilenberg & Hoogstraal, 1969

Endemic to Madagascar, this species has been collected in different areas, including the north and northwest. It is known to parasitize introduced rodents and medium-size furred tenrecs [32]. No details currently available associated with its role as a pathogen vector.

#### *Ixodes simplex* Neumann, 1906

This tick is a bat parasite across different portions of Africa, Europe, and Asia. On Madagascar, adults and the immature stages of this species have been collected from different bats, including *Miniopterus* spp. and *Myotis goudoti* [47]. Its potential role as a pathogen vector is unknown.

#### *Ixodes soarimalalae* Apanaskevich & Goodman, 2020

Endemic to Madagascar, it was identified from collections made in the eastern part of the island (Atsimo Atsiananana Region) and at elevations over 800 m, where it parasitizes small furred tenrecs [92]. Nothing is known about pathogen transmission of this tick.

#### *Ixodes uilenbergi* Apanaskevich & Goodman, 2020

Endemic to Madagascar, this species has been found in the Central Highlands and in portions of the east between 980 and 1990 m above sea level [92]. It is known to parasitize spiny and furred tenrecs. Currently, its role as a vector of pathogens is undocumented.

#### *Ixodes uncus* Apanaskevich & Goodman, 2020

Endemic to Madagascar, this species was recently described from furred tenrecs collected in the Central Highlands at elevations of 1300–1600 m above sea level [92]. No details currently available associated with its role as a pathogen vector.

#### Genus *Rhipicephalus* Koch, 1844

The genus is found worldwide, in most tropical and subtropical regions, as well as in more temperate region, parasitizing mainly mammals. Of the 90 species described worldwide [31], four species are found in the SWIO region.

#### *Rhipicephalus appendiculatus* Neumann, 1901

This “cattle tick” commonly known as the brown ear tick is widely distributed across sub-Saharan Africa and is a vector of *Theileria parva*, the causative organism of East Coast fever and Corridor disease in cattle [93]. *Rhipicephalus appendiculatus* also transmits benign bovine theileriosis caused by *T. taurotragi* [94]. Established populations have been recorded on Grande Comore (Comoros Archipelago), where it is particularly abundant [58, 59]. It has also been reported on Mauritius [56]. Records from eastern Madagascar, specifically the port of Toamasina, indicate that the species was introduced through cattle imports from ports from South Africa but has not succeeded in establishing viable populations [32]. Genetic analysis reveals that the Grande Comore population of this tick belongs to the Southern clade [58], a lineage known for its high vector competence for *T. parva*, the agent of East Coast fever in cattle. Furthermore, studies in the Comoros detected *Rickettsia africae* in this tick [64]. The abundance of *Rhipicephalus appendiculatus* in the Grande Comores is positively correlated with the prevalence of *T. parva* and *Ehrlichia ruminantium* antibodies in cattle, goat, and sheep, underscoring its epidemiological importance in the region [59].

#### *Rhipicephalus evertsi* Neumann, 1897

This species commonly known as the red-legged tick is widely distributed across sub-Saharan Africa, where it mostly parasitizes cattle, horses, and other ungulates [95, 96]. *Rhipicephalus evertsi* is considered as a vector of the Crimean Congo Hemorrhagic Fever virus [97] and an important vector of *Theileria equi*, the cause of equine piroplasmosis, and *T. separata*, the cause of ovine theileriosis, as well as, *Babesia caballi* affecting horses. The saliva of engorging female ticks contain a toxin that causes paralysis, particularly in young sheep, but it may also affect cattle calves and adult sheep [96]. While absent from most of the landmasses in the SWIO region, it has become established on Mauritius, where it is a potential vector of *B. equi* for horses and *B. ovis* and *T. ovis* for small ruminants [56].

#### *Rhipicephalus microplus* (Canestrini, 1888)

This cosmopolitan species, known as the Asian blue tick, is widely distributed and established across different areas of the world and is currently prevalent in diverse habitats across all SWIO islands [57]. It has been postulated that this tick species was brought to eastern and southern Africa from Madagascar through live animal trade, where it was initially introduced via cattle imported from southern Asia [98]. It parasitizes mainly cattle but also sheep, goat, horses, deer, dogs, and occasionally bites humans [32]. This tick transmits horizontally and/or vertically bovine babesiosis (*Babesia bovis* and *B. bigemina*) and bovine anaplasmosis (*Anaplasma marginale*) [99, 100]. In the Comoros, this species is the most frequently recorded tick on Grande Comores, Anjouan, and Mohéli [58]. Populations of *R. microplus* from Madagascar have shown variable levels of resistance to arsenic, to hexachlorocyclohexane (HCH), and other organochloride insecticides [101]. On La Réunion, populations of *R. microplus* exhibited differing levels of susceptibility to the acaricidal effects of several selected plants, including *Peperomia borbonensis* (Piperaceae), *Zanthoxylum heterophyllum* (Rutaceae), *Monimia rotundifolia* (Monimiaceae), and *Psiadia amygdalina* (Asteraceae) [102]. On Madagascar, *R. microplus* is a potential vector of multiple pathogens, including *A. marginale* the agent of bovine anaplasmosis [23], *Borrelia* sp. (likely *B. theileri* the cause of spirochetosis in cattle), *Ehrlichia ruminantium*, *Rickettsia africae*, *Coxiella burnetii* [48, 66, 67, 103] and the Crimean–Congo hemorrhagic fever virus [104], which was found to be phylogenetically close to isolates from Asian countries [105]. In addition, in the Comoros (Grande Comores and Anjouan), *Rhipicephalus microplus* is also a potential vector of *Rickettsia africae* [64]. On La Réunion, this species is involved in the transmission of *Babesia bovis* and *B. bigemina* [65]

#### *Rhipicephalus sanguineus* sensu lato (Latreille, 1806)

This species is widely distributed around the world, generally parasitizing dogs [106]. In the SWIO region, it is reported to be present on La Réunion, Madagascar, and Mauritius [32, 56, 107]. On Madagascar, it is suspected to be a vector of *Babesia canis*, although there has been no local experimental evidence of transmission [32].

#### Genus *Sharifiella* Dias, 1958

This genus was originally assigned to the genus *Haemaphysalis*, but a recent revision based on morphological characters and molecular analyses resulted in the elevation of *Sharifiella* to a full genus [108]. It is currently monotypic, comprising a single species that occurs in the SWIO region.

#### *Sharifiella theilerae* (Hoogstraal, 1953)

Endemic to Madagascar, this species is found in both the north, the south, the southeast of the island, as well as the Central Highlands, parasitizing spiny tenrec species [32, 72]. Its role as a pathogen vector is unknown.

### Circulation of tick-borne diseases (TBDs) in the SWIO region

Ticks can serve as vectors for a broad range of infectious agents, encompassing bacteria, viruses, and parasite protozoa. The majority of TBDs are of zoonotic origin, maintained by cycles involving wildlife and domestic animal reservoirs where the infectious agents multiply and persist. Humans are typically accidental hosts and usually represent a dead end for the infectious agent. In the SWIO region, several investigations have recorded the circulation of different tick-borne diseases and the associated pathogens. These are listed below in alphabetic order.

#### African swine fever

African swine fever (ASF) is a viral disease of Suidae, caused by a DNA virus of the family Asfarviridae, genus *Asfivirus*. ASF has spread across Africa, Europe, and Asia, severely challenging domestic pig-producing countries and negatively affecting trade in pork products [109]. In the SWIO region, ASF was first suspected clinically and later confirmed on Madagascar in 1998. Since then, the virus has disseminated to almost all pig-raising areas on the island, causing significant economic losses [110, 111]. Further, on Madagascar the introduced African bushpig (*Potamochoerus larvatus*) is unlikely to play a significant role in the maintenance or transmission of ASF, whereas *Ornithodoros moubata* ticks may be capable of sustaining ASF virus circulation within domestic pig populations [112]. On Mauritius, an ASF outbreak was detected in 2007, most likely introduced from Madagascar or mainland Africa; this led to the implementation of stringent biosecurity measures, which ultimately achieved disease eradication [113, 114].

#### Anaplasmosis

Bovine anaplasmosis, caused by the obligate intracellular bacterium pathogen *Anaplasma marginale*, is prevalent in tropical and subtropical regions, and significantly impacts cattle production in many countries [115, 116]. *Anaplasma marginale* is transmitted by over 20 tick species [115], two of which (*Ambylomma variegatum* and *Rhipicephalus microplus*) are present in the SWIO region. On Madagascar, this pathogen was first detected in cattle in 1965 using the splenectomy method [25]. More recently, a high prevalence of *Anaplasma marginale* has been confirmed in cattle through PCR and sequencing techniques [23]. Strains identified on Madagascar clustered with those isolated in South Africa, America, and Israel [23]. Another study highlighted the presence of *Anaplasma* antibodies in the domestic ruminant populations (cattle, goats, and sheep) in the Comoros, as well as in cattle on La Réunion, underscoring the circulation of the pathogen among livestock [24, 59].

Canine anaplasmosis, another TBD of global significance, involves different *Anaplasma* species, such as *A. platys* or *A. phagocytophilum* [117]. On Mauritius, *Anaplasma* antibodies have been detected in dogs through serological studies [107].

#### Babesiosis

This disease is caused by infection with intraerythrocytic protozoan parasites of the genus *Babesia.* Babesiosis is one of the most widespread infections affecting free-living animals worldwide and is the subject of increasing interest as an emerging zoonosis in humans [118]. Although capable of infecting a wide range of vertebrates, *Babesia* parasites require both a competent vertebrate host and non-vertebrate vector to maintain transmission cycles. All *Babesia* parasites described to date are transmitted by Ixodidae ticks to their vertebrate hosts [119]. In the SWIO region, at least eight Ixodidae species (three *Rhipicephalus* species and five *Haemaphysalis* species) have been identified as potential vectors of *Babesia*. On Madagascar, *Babesia* parasites have been found to be particularly prevalent in wild lemurs, as revealed by PCR and sequencing analyses [90], while *B. bovis* (historically referred to as *B. argentina*) and *B. bigemina* have been detected in cattle using splenectomy methods [25]. In the Comoros, the circulation of Babesiosis has been confirmed through the detection of *B. bigemina* antibodies in the serum of cattle, goats, and sheep [22, 59]. On La Réunion, both *B. bigemina* and *B. bovis* have been reported in cattle [24, 56], as is the case for *B. bovis* on Mauritius [56].

#### Borreliosis

Infectious bacteria of the genus *Borrelia* include a diverse group of spirochetes that have a nearly global distribution. Transmitted by ticks from the Argasidae and Ixodidae families, these spirochetes have an obligate enzootic life cycle involving various vertebrate hosts, including mammals, reptiles, and seabirds [120, 121]. In the SWIO region, five tick species (*Ornithodorus capensis*, *Ambylomma variegatum*, *Haemaphysalis lemuris*, *H. simplex*, and *Rhipicephalus microplus*) are potential vectors of *Borrelia* bacterium. On Madagascar, *Borrelia* has been detected in the serum of several lemur species through PCR and sequencing analyses, with infections appearing to be less common during the summer months [90]. Historically, *B. duttoni* was recorded in the western part of Madagascar until 1950 [122], after which it seems to have disappeared for unknown reasons [123]. In addition, *B*. *theileri* has been detected in cattle on La Réunion and Mauritius based on blood smears [56].

#### Cowdriosis

Also known under different names, such as Ehrlichiosis and heartwater, Cowdriosis is a fatal disease affecting domestic and wild ruminants. Caused by the bacterium *Ehrlichia ruminantium* and transmitted by ticks of the genus *Amblyomma*, it is a significant cause of mortality in cattle, sheep, and goats in tropical and subtropical regions [124]. This disease is known to occur in Africa and several localities in the SWIO region [125], where three tick species (*A. variegatum*, *Rhipicephalus appendiculatus*, and *R. microplus*) are considered potential vectors. Previous investigations have demonstrated the circulation of *Ehrlichia* bacteria in the Comoros (including Mayotte), and on La Réunion and Mauritius [22, 24, 59, 107, 126]. Specifically, in the Comoros, serological studies on bovine, goat, and sheep populations have revealed the presence of *Ehrlichia* antibodies in their serum [22, 59]. Similarly, antibodies against *Ehrlichia* have been detected in dogs and an introduced deer (*Cervus timorensis*) from Mauritius [107, 127], as well as in cattle from La Réunion [24].

#### Crimean Congo hemorrhagic fever (CCHF)

This widespread disease is caused by a virus transmitted to humans primarily through the bite of Ixodidae ticks, particularly those of the genus *Hyalomma.* Transmission can also occur through crushing infected ticks, contact with the blood or tissues of infected animals, or exposure to the bodily fluids of a patient with CCHF during the acute phase of infection. On Madagascar, serological surveys conducted between 2008 and 2009 among slaughterhouse workers revealed evidence of CCHF virus circulation in the human population, although the prevalence of immunoglobulin G (IgG) and immunoglobulin M (IgM) was low [27].

#### Hepatozoonosis

Canine Hepatozoonosis, with nearly worldwide distribution, is an animal disease caused by the protozoan parasite *Hepatozoon canis*, which is transmitted to dogs by various Ixodidae tick species including *Rhipicephalus sanguineus* sensu lato [128]; the latter being present in the SWIO region. Such infections have been identified on Mauritius, confirmed through the observation of parasitized neutrophils and by PCR sequencing of canine blood samples [107, 129].

#### Neoehrlichiosis

Neoehrlichiosis is an emerging and expanding TBD, currently reported mainly in Europe and parts of Asia. It is caused by bacteria of the genus *Neoehrlichia*, particularly *N. mikurensis*, which is transmitted by Ixodidae ticks and maintained in enzootic cycles involving rodents as reservoir hosts [130]. However, the known Ixodidae vector species, including *Ixodes ricinus*, *I. persulcatus*, *I. ovatus*, *I. frontalis*, *I. hexagonus*, *Dermacentor reticulatus*, and *Haemaphysalis concinna* have not been documented in the SWIO region. It can be pathogenic to humans, causing a systemic inflammatory syndrome that mostly affects individuals with underlying hematologic or autoimmune disorders [131]. Although research on the circulation of *Neoehrlichia* in the SWIO remains limited, a study on Madagascar detected *Neoehrlichia* DNA in different lemur species using PCR and sequencing techniques [90].

#### Q Fever disease

Q fever occurs nearly worldwide, with some exceptions, such as New Zealand and French Polynesia [132]. The disease is caused by the bacterium *Coxiella burnetii*, whose primary reservoir is mammals, such as sheep, goats, and cattle, and with transmission to humans occurring mainly through inhalation of contaminated aerosols or direct contact with infected animals. The role of ticks in the epidemiology of Q fever remains debated, as *C. burnetii* is only rarely detected in ticks [133]. In the SWIO region, four tick species has been identified as potential vectors of this bacterium: *Amblyomma variegatum*, *Ornithodoros capensis*, *Otobius megnini*, and *Rhipicephalus microplus*. On Madagascar, anti-*C. burnetii* antibodies have been detected in cattle serum samples, indicating exposure to the pathogen in local livestock populations [66]. On La Réunion, the circulation of the *C. burnetii* in humans and ruminants has also been observed [134, 135].

#### Spotted fever rickettsiosis

Spotted fever rickettsioses are globally distributed zoonotic diseases caused by several bacterial species belonging to the genus *Rickettsia*. They are primarily transmitted to humans through the bites of infected ticks, with rodents and other vertebrates serving as reservoir hosts [136]. In the SWIO region, multiple *Rickettsia* species have been detected in ticks, and at least nine tick species have been identified as potential vectors. Among these, *R. conorii*, one of the causative agents of spotted fever, has been circulating on Madagascar for over a century. Infections of this bacterium were first reported in the western and eastern regions of the island during the early 1900s, based on serological studies conducted of human and animal serum samples [137]. More recently, a serological study on human plasma samples from pregnant women collected across various localities and altitudinal zones of Madagascar revealed *R. conorii* seropositivity [26]. On La Réunion, approximately one-fifth of the human population was found to have antibodies reactive to at least one *Rickettsia* species, including members of the spotted fever group [138].

#### Theileriosis

Bovine theileriosis, is a group of tick-borne diseases caused by protozoan parasites of the genus *Theileria* [139]. Economically important *Theileria* species that infect cattle and small ruminants are transmitted by Ixodidae ticks. Among them, *T. parva*, the causative agent of East Coast Fever, whose the main tick vector, *Rhipicephalus appendiculatus* is present in the Comoros, is the most pathogenic species in Africa, causing high morbidity and mortality [93]. On Grande Comore, antibodies against *T. parva* have been detected in the sera of bovine [59] and the pathogen has also been detected using molecular techniques in the lymph node biopsies of bovine [22]. In addition, *T. mutans* has been detected in cattle of Madagascar through splenectomy methods [25]; however, this parasite generally causes little or no illness.

#### Tick-borne encephalitis

Tick-borne encephalitis (TBE) is a viral infection of the central nervous system transmitted to humans in Europe and Asia primarily through the bite of different Ixodidae species [140]; these tick species have not yet been documented in the SWIO region. However, a serological study on Grande Comores have provided evidence of TBE circulation among humans, although the seropositive prevalence remains low [141].

### Overview of ticks and TBDs

In summary, the information provided herein indicate that ticks and TBDs are widely distributed across the SWIO region, affecting humans, livestock, companion animals, and wildlife. Although the documented tick fauna differs markedly among islands, several pathogens have been detected repeatedly in the region, either in vertebrate hosts or in associated tick species. For some pathogens, their detection in the region has only been reported on islands where the corresponding tick vectors have been documented, whereas no reports are available from islands where these vectors are absent. This pattern is illustrated by African swine fever virus, whose recognized tick vector is present on Madagascar but has not been reported from other portions of SWIO. In contrast, pathogens belonging to the genera *Anaplasma*, *Ehrlichia*, *Babesia*, and *Borrelia* show evidence of sustained or historical circulation across multiple islands in the region and a variety of hosts.

## Discussion

This present review provides a comprehensive summary of current knowledge on the diversity of ticks and the circulation of TBDs in the SWIO region.

### Diversity of tick species and their consequence on human and animal health across the region

The ticks in the SWIO region represent approximately 4% of the 1033 species described worldwide [31]. Overall, the regional tick fauna is characterized by both low species richness and high endemism, a pattern consistent with classical island biogeography. To date, 42 tick species have been documented across the SWIO, with the vast majority recorded on Madagascar (38 species), compared to significantly fewer species on smaller and more isolated islands such as La Réunion, Mauritius, Rodrigues, the Comoros, the Seychelles, and the Eparses Islands. This uneven distribution is consistent with an insular effect, whereby island size, isolation, habitat diversity, and host availability limit species richness, particularly on small islands. At the same time, the high proportion of endemic species–all of which are restricted to Madagascar, with no endemic ticks reported on the other islands–suggests long-term isolation and local diversification.

Nevertheless, the low number of species currently recorded is likely influenced not only by biogeographical constraints but also by limited research. Indeed, beyond the expected effects of island size discussed above, research and taxonomic efforts on ticks have been limited, with most species having been described in the 1900s [87, 142–144] and eight species named since the 2000s [73, 85, 91, 92, 108]. We hypothesize that increased sampling efforts and systematic studies, particularly with molecular tools, will lead to new discoveries, specifically on Madagascar, where the percentage of tick endemicity is high–around 79% of the species recorded in the SWIO region are endemic to this island. Most importantly, given the key role of native terrestrial mammals in tick diversification and the fact that, outside Madagascar, the only native mammals on the other islands are bats, the discovery of previously undescribed tick species is expected to predominantly concern Madagascar. However, on the other islands of the SWIO region, additional findings cannot be excluded. Therefore, efforts to train systematists specializing in ticks and to refine the tick taxonomy of the region are essential. Some of the previously unrecognized species might be important in the transmission of tick-borne pathogens. In parallel, taxonomic issues relating to endemic ticks could be addressed using molecular tools to identify them, especially larvae and nymphs, which are morphologically difficult to determine at the species level.

From an evolutionary perspective, the phylogenetic origin of the SWIO region ticks remains poorly resolved, largely due to the scarcity of molecular studies, especially on endemic taxa. On a broader geographical scale, molecular clock analyses based on mitochondrial genomes indicate that Ixodidae ticks originated in the late Paleozoic (~ 319 Ma) and that major family‑level divergences (e.g., Argasidae vs. Ixodidae) occurred in the Permian (~ 290 Ma) and Triassic (~ 249 Ma) [145]. Although no comprehensive molecular clock analyses have yet been conducted specifically for Malagasy or SWIO ticks, analyses based on 18S rDNA sequences have suggested an emergence of *Haemaphysalis lemuris*, an endemic Malagasy species, whose origin, according to divergence estimates, dates back to the Cenozoic, less than 20 million years old [146]. Its emergence therefore postdates the radiation of its principal hosts, lemurs, which is placed in the early to middle Eocene, around 50 million years ago, based on nuclear gene analyses [147]. This finding implies that the evolutionary history of at least this endemic tick species likely reflects more recent diversification processes. Future phylogenomic studies integrating SWIO taxa into global tick datasets and applying robust molecular dating methods will be essential to clarify the extent to which tick diversity in the region is due to landmasses breaking apart, long-distance tick dispersal, or ticks evolving with specific hosts.

Among the tick species recorded in the SWIO region, 17 (approximately 41%) are known or considered as potential vectors of pathogens. This estimate is based either on the detection of pathogens in field-collected ticks or on epidemiological evidence showing a positive association between tick abundance and pathogen prevalence, even when experimental transmission has not been demonstrated. In addition, four species, namely – *Amblyomma variegatum*, *Rhipicephalus appendiculatus*, *R. microplus*, and *R. sanguineus* sensu lato – have been experimentally confirmed as vectors of pathogens in other parts of the world. For instance, *R. appendiculatus* has been shown to transmit *Theileria parva* [93]. This parasite was responsible for the East Coast Fever epidemic in 2002–2004 on Grande Comore and led to the death of 10% of cattle on that island [22]. *Amblyomma variegatum* can transmit *Rickettsia africae* through both transovarial and trans-stadial transmission [148] and is capable of transmitting *Ehrlichia ruminantium* [149]. *Rhipicephalus sanguineus* sensu lato has been shown to transmit pathogens such as *Anaplasma marginale*, *Rickettsia conorii*, *Ehrlichia canis*, *Coxiella burnetii*, and *Hepatozoon canis* through transstadial transmission and/or direct transmission to the host [150, 151]. Furthermore, *Rhipicephalus microplus* has been shown to acquire *Babesia bovis* and *Anaplasma marginale* and may maintain infection across developmental stages, although the role of transovarial transmission for *A. marginale* remains debated [152, 153].

All these pathogens are known to circulate across the SWIO region. Consequently, the presence of these tick species, along with the pathogens they carry and transmit, pose significant risks to human and animal health in the region. However, differences in vector competence can arise depending on the vector populations or the specific pathogens used during experimental infections [154]. Thus, further research is essential to confirm the role of these species in the transmission of tick-borne pathogens in the region. A major challenge in conducting these studies lies in replicating a complete transmission cycle under experimental conditions. Among other logistical and technical constraints, this requires maintaining tick colonies, having access to vertebrate hosts that are compatible with both the ticks and pathogens, developing efficient artificial tick feeding techniques, culturing pathogens, and working in high-biosafety-level facilities.

Among the pathogens circulating in the region, Crimean Congo Hemorrhagic Fever (CCHF) stands out as one of the most concerning human TBDs. It is one of the most widely distributed Old World tick-associated diseases globally, with a range extending from southern Russia and the Black Sea region to the southern tip of Africa [155]. The disease has an annual and periodic human fatality rate of approximately 10% of reported cases [156]. Although *Hyalomma marginatum*, the primary vector of this arbovirus [157], is absent from the SWIO islands, the virus has been isolated from field collections on Madagascar of *Rhipicephalus microplus* [104]; however, the role of this species in CCHF transmission should be further investigated. In addition, CCHF antibodies have been observed in at-risk professionals on Madagascar, such as those working in slaughterhouses, handling fresh meat or coming in contact with livestock blood, although the infection rate in this region is relatively low compared to countries where CCHF is endemic, like in Mauritania and the United Arab Emirates [27].

The biting behavior of certain tick species could play a significant role in the (re)emergence of TBDs in the SWIO region. Seven species–*Ornithodoros porcinus*, *Otobius megnini*, *Ambylomma variegatum*, *A. loculosum, Haemaphysalis elongata*, *H. lemuris*, and *Rhipicephalus microplus*–are known to feed primarily on wildlife and domestic animals, but can also bite humans, at least during their immature stages [32]. Among them, the exophilic ticks *A.variegatum* and *R. microplus* can act as potential bridge vectors, transmitting pathogens between animals and humans; thus, increasing the risk of zoonotic disease transmission.

Moreover, the SWIO islands, especially Madagascar and the Comoros, are experiencing high rates of deforestation, leading to the rapid encroachment of anthropogenic areas into natural habitats [158, 159]. In turn, a large portion of Mauritius was cleared of its natural forest cover since nearly a century [160]. This overlap between human settlements and forested regions disrupts vector-host dynamics and increases the potential for human contact with sylvatic reservoirs of zoonotic pathogens [161]. As a result, opportunistic tick species can serve as bridge vectors, facilitating the transfer of pathogens between sylvatic and rural/urban transmission cycles. This could in turn facilitate the (re)emergence of TBDs at the regional or beyond. Indeed, historically, the SWIO region played a key role in human-mediated animal movement, particularly livestock, between east Africa, the Middle East, and southwest Asia. These movements increase the risk of amplifying and spreading diseases on a global scale. Consequently, the role of tick species as bridge vectors in both rural and urban environments, especially in the context of changing land-use patterns, should be a key focus of future research and surveillance efforts.

### Risks of introduction and spread of TBDs in the SWIO region linked to animal movement and livestock trade

Connections between the SWIO islands and other regions, particularly Africa and Asia, have occurred for centuries, primarily driven by human migration and trade. The introduction and circulation of domestic animals, especially cattle, played a significant role in these exchanges. Genetic studies indicate that the cattle of Madagascar and Comoros originated from a mixture of African and Indian zebu, a crossbreeding dating from the twelfth century [162]. This coincided with early interactions between African populations along the Swahili corridor and Austronesians from southeast Asia in the Comoros and Madagascar [162]. Later, in the seventeenth and eighteenth centuries, Malagasy zebu were introduced to Mauritius and La Réunion [163]. Beyond domestic animals, wildlife species were also displaced, including spiny tenrecs (*Tenrec ecaudatus*), bush pigs (*Potamochoerus*), and tortoises (several different genera), which were moved between the east African coast and its offshore islands (such as Mafia, Zanzibar, and Pemba) and SWIO islands [164–166], particularly Madagascar and the Comoros [167].

These human and animal migrations have contributed to the spread of ticks across the SWIO region. On Indian Ocean islands the introduction of the cattle ticks *Ambylomma variegatum* and *Rhipicephalus microplus* presumably coincided with the arrival of their hosts following human settlement. *Amblyomma variegatum*, originally from east Africa, was reported on Madagascar and Mauritius in 1899 and later on La Réunion in 1949 [32, 56]. As for* R. microplus*, which originated in southeast Asia, its global dispersal was facilitated by colonial-era cattle transportation [168]. It was first documented on Madagascar by Minning in 1934, though it was likely introduced to the island around the same time as *A. variegatum* from Africa [168]. The Mascarenes were probably infested from Madagascar through cattle introductions, with *R. microplus* being reported on Mauritius in 1915 and on La Réunion in 1949 [169, 170].

*Otobius megnini*, the spinose ear tick, was first detected on Madagascar in 1948 [32]. It was introduced from South Africa and may have evaded monitoring in Toamasina during cattle importation [32]. Similarly, *R. appendiculatus* was introduced to the Comoros in 2002 through cattle imports from Tanzania and is now well established on Grande Comore [57]. This tick species was also introduced to Madagascar via cattle imports from South Africa, but it failed to establish a permanent population.

At least six other tick species–*A. hebraeum*, *Hyalomma rufipes*,* H.truncatum*, *Margaropus winthemi*,* R. capensis*, and *R. evertsi*–were also introduced to Madagascar through cattle importation from South Africa, but failed to establish [32]. Specifically, *H. truncatum* and *R. evertsi* arrived in 1963 via goat imports from South Africa. These goats were not quarantined upon arrival in Toamasina and were transported directly to Ambovombe in the south, where the ticks were detected [32]. In addition, *R. pulchellus* was found on Madagascar on cattle imported from Kenya, while *Ixodes canisuga* was observed on a dog imported from France. However, none of these species became established on the island [32].

This historical pattern of animal introductions highlights the risks associated with importing animals into the SWIO region, especially in the absence of proper quarantine and surveillance measures. The repeated introduction of tick species–some of which became established, while others did not–demonstrates how human-mediated animal movements can facilitate the spread of ectoparasites, as well as their associated pathogens. For instance, in the Comoros, the epidemic of East Coast Fever emerged shortly after cattle were imported from Tanzania, and researchers found that *Theileria parva* parasites isolated in the Comoros had identical genetic profiles to those used in the cocktail vaccine (a live vaccine containing a combination of several *T. parva* strains to induce broad immunity) administered on a large scale in Tanzania [22]. It is likely that some of the imported cattle had been vaccinated with the cocktail vaccine and subsequently became asymptomatic carriers of the disease [171]. As the pathogen spread rapidly across the island, researchers hypothesized that *R. appendiculatus*, a tick species also introduced from Tanzania, became infected by feeding on the carrier cattle [22]. The infected ticks then transmitted the disease to local immunologically naive cattle populations, exacerbating the outbreak. Subsequent research has highlighted a high prevalence of *Rickettsia africae* infection in *A. variegatum* collected from cattle imported from Tanzania to the Comoros [64].

The African Swine Fever virus was also introduced into Madagascar, likely between 1997 and 1998, through imports from east Africa [110, 113]. Genetic analysis of the isolated virus revealed a close similarity with strains from Mozambique [110]. The virus spread across the island, with the exception of the northern and western regions. The animal responsible for the introduction of this virus remains unidentified due to the lack of an effective surveillance system. The disease has also been introduced in Mauritius in 2007 [113]. Introductions of non-native species, such as the rusa deer (*Cervus timorensis rusa*) from Indonesia to Mauritius in the sixteenth century, have similarly exposed islands to new pathogens, including *Ehrlichia* species [127]. Similar patterns have been reported for other livestock diseases, further supporting the role of cattle movements between mainland Africa and the SWIO region. In particular, the introduction and recurrent outbreaks of Rift Valley fever in the SWIO region have been linked to the importation of infected livestock from east Africa, as well as to intra-island cattle movements [172–174], which, once again, highlights the epidemiological risks associated with insufficient animal movement control and surveillance.

Although the dispersal of TBDs across the SWIO islands via small mammals remains poorly documented, this pathway warrants closer attention. *Tenrec ecaudatus*, a terrestrial mammal endemic to Madagascar, has been deliberately displaced from Madagascar to several islands in the region. These introductions involved all four islands of the Comoros as well as Mauritius, La Réunion, and several islands of the Seychelles (Mahé, Praslin, Thérèse, and Anonyme). Archaeological and historical evidence indicates that the earliest introduction occurred through long-distance transfer, with the arrival of *Tenrec ecaudatus* in the Comoros (Mayotte) dating back to the eighth century [175, 176]. Subsequent introductions took place in the Mascarenes during the late eighteenth century in Mauritius and the early nineteenth century in La Réunion [177], followed by introductions to the Seychelles, apparently around 1880 [178, 179]. Morphological characteristics of this species particularly the presence of dense spines may facilitate the persistence of ectoparasites, as ticks can attach and remain concealed in areas that are difficult for the host to groom effectively. Although no published evidence currently documents the introduction of tick species or TBDs from Madagascar to other Indian Ocean islands via the translocation of *Tenrec ecaudatus*, this species has been identified as a reservoir of other zoonotic pathogens [180, 181]. Its extensive history of human-mediated dispersal, combined with its ecological adaptability and potential to host important number of ticks, suggests that its role in the regional circulation of parasites and associated pathogens warrants further investigation.

Beyond the Indian Ocean and African regions, the circulation of small mammals from the SWIO region and their ticks has contributed to the dispersion of pathogens [182]. Reptiles and amphibians exported from Madagascar to Japan were found to be positive for *Rickettsia* bacteria, which was phylogenetically close to the *Rickettsia* strain isolated from the United States [182]. However, given that these animals may have come into contact with other hosts and tick species during transport and after arrival in Japan, a direct link between Malagasy animals and this *Rickettsia* isolate remains inconclusive.

In addition to human-mediated animal movements, the natural dispersal of ticks and TBDs without direct human assistance should also be considered, particularly in insular environments. Migratory birds may play a significant role in the dispersal of these ectoparasites and their associated pathogens, a pathway that is considerably more difficult to manage than the spread of harmful organisms via trade routes and human travel [183]. Migratory seabirds, in particular, are well recognized as long-distance dispersal agents for ticks, facilitating their movement across continents and to remote oceanic islands during migration. Tick species associated with these seabirds (*Amblyomma loculosum* and *Carios capensis*) have been documented in the SWIO region which have been identified as potential or confirmed vectors of different pathogens [34, 35, 38]. Although these bird-associated ticks are generally considered to play a limited role in the transmission of pathogens to domestic animals and humans due to their restricted contact with these hosts, their presence nevertheless highlights the capacity of ticks to colonize islands independently of human activities.

In light of these historical introductions and the circulation of ticks and pathogens into the SWIO region, it is crucial to strengthen surveillance, quarantine, and biosecurity measures. The repeated emergence of infectious diseases, often facilitated by uncontrolled animal importations and intra-island movements, underscores the vulnerabilities of island ecosystems to invasive species [184]. Effective monitoring systems, early detection strategies, and coordinated regional inter-governmental policies are essential to prevent future outbreaks and mitigate their economic and ecological impacts. Strengthening veterinary control systems and enforcing stricter import rules can help limit the spread of diseases and protect both animals and humans.

## Conclusions

Ticks and tick-borne diseases represent a significant threat to both human and animal health in the SWIO region. Strengthening veterinary infrastructure, taxonomic work on these ectoparasites, enhancing surveillance systems, and promoting collaboration among stakeholders and regional governments will be essential to prevent future outbreaks. Addressing these challenges underscores the critical importance of a One Health approach. Furthermore, expanding research on ticks and tick-borne diseases in the region is crucial for improving prevention, control and response efforts.

## Data Availability

Data supporting the main conclusions of this study are included in the manuscript.

## References

[1] McCoy KD, Boulanger N. Tiques et maladies à tiques : biologie, écologie évolutive, épidémiologie. Marseille: IRD Edition; 2015.

[2] Camicas J-L, Hervy J-P, Adam F, Morel PC. Les tiques du monde (Acarida, Ixodida): nomenclature, stades décrits, hôtes, répartition. Paris: ORSTOM edition; 1998.

[3] Dietrich M, Gómez-Díaz E, McCoy KD. Worldwide distribution and diversity of seabird ticks: implications for the ecology and epidemiology of tick-borne pathogens. Vector Borne Zoonotic Dis. 2011;11:453–70. 10.1089/vbz.2010.0009. 20874222

[4] Descamps S. Winter temperature affects the prevalence of ticks in an Arctic seabird. PLoS ONE. 2013;8:1–5. 10.1371/journal.pone.0065374 PMC3672161 23750259

[5] Peñalver E, Arillo A, Delclòs X, Peris D, Grimaldi DA, Anderson SR, et al. Ticks parasitised feathered dinosaurs as revealed by Cretaceous amber assemblages. Nat Commun. 2017;8:1–12. 10.1038/s41467-017-01550-z PMC5727220 29233973

[6] Larson G, Fuller DQ. The Evolution of animal domestication. Annu Rev Ecol Evol Syst. 2014;66:115–36.

[7] Jongejan F, Uilenberg G. The global importance of ticks. Parasitology. 2004;129:S3-14. 10.1017/s0031182004005967 15938502

[8] Durden L. Taxonomy, host associations, life cycles and vectorial importance of ticks parasitizing small mammals. In: Micromammals and macroparasites from evolutionary ecology to management. Japan: Springer; 2006. p. 91–102.

[9] Radolf JD, Strle K, Lemieux JE, Strle F. Lyme disease in humans. In: Curr issues mol biol, vol. 42. Caister Academic Press; 2020. p. 1–83. 10.21775/cimb.042.333 PMC7946767 33303701

[10] Elbir H, Raoult D, Drancourt M. Review article: relapsing fever borreliae in Africa. Am J Trop Med Hyg. 2013;89:288–92. 10.4269/ajtmh.12-0691 PMC3741250 23926141

[11] Gilbert L. The impacts of climate change on ticks and tick-borne disease risk. Annu Rev Entomol. 2021;66:373–88. 10.1146/annurev-ento-052720-094533 33417823

[12] Hoellinger B, Parfut A, Grisard M, Martin-Latil S, Denis J, Augereau O, et al. Tick-borne encephalitis: an ancient pathology, but a current emergence in Europe. Infect Dis Now. 2025;56:105187. 10.1016/j.idnow.2025.105187 41242395

[13] Ergönül Ö. Crimean-Congo haemorrhagic fever. Lancet Infect Dis. 2006;6:203–14. 10.1016/S1473-3099(06)70435-2 PMC7185836 16554245

[14] Wu YX, Yang X, Leng Y, Li JC, Yuan L, Wang Z, et al. Human-to-human transmission of severe fever with thrombocytopenia syndrome virus through potential ocular exposure to infectious blood. Int J Infect Dis. 2022;123:80–3. 10.1016/j.ijid.2022.08.008 35987469

[15] Kasaija PD, Estrada-Peña A, Contreras M, Kirunda H, de la Fuente J. Cattle ticks and tick-borne diseases: a review of Uganda’s situation. Ticks Tick Borne Dis. 2021;12:1–10. 10.1016/j.ttbdis.2021.101756 34134062

[16] Eskezia BG, Desta AH. Review on the impact of ticks on livestock health and productivity. J Biol Agric Health. 2016;6:1–7.

[17] Goodman SM. Updated estimates of biotic diversity and endemism for Madagascar-revisited after 20 years. Oryx. 2023;57:561–5.

[18] Goodman SM, Soarimalala V. Introduction to mammals. In: Goodman SM, editor. The new natural history of Madagascar. Princeton: Princeton University Press; 2022. p. 1737–69.

[19] Cardiff SG, Jenkins RKB. The bats of Madagascar: a conservation challenge. Lessons Conservation. 2016;6:80–108. 10.5531/cbc.linc.6.1.6.

[20] Christidis L, Goodman SM, Naughton K, Appleton B. Insights into the evolution of a cryptic radiation of bats: dispersal and ecological radiation of Malagasy *Miniopterus* (Chiroptera: Miniopteridae). PLoS ONE. 2014;9:1–10. 10.1371/journal.pone.0092440 PMC3958536 24642892

[21] O’Brien J. Bats of the Western Indian Ocean Islands. Animals. 2011;1:259–90. 10.3390/ani1030259 PMC4513465 26486500

[22] De Deken R, Martin V, Saido A, Madder M, Brandt J, Geysen D. An outbreak of east coast fever on the Comoros: a consequence of the import of immunised cattle from Tanzania? Vet Parasitol. 2007;143:245–53. 10.1016/j.vetpar.2006.08.018 16996692

[23] Pothmann D, Poppert S, Rakotozandrindrainy R, Hogan B, Mastropaolo M, Thiel C, et al. Prevalence and genetic characterization of *Anaplasma marginale* in zebu cattle (*Bos indicus*) and their ticks (*Amblyomma variegatum, Rhipicephalus microplus*) from Madagascar. Ticks Tick Borne Dis. 2016;7:1–8. 10.1016/j.ttbdis.2016.08.013 27592064

[24] Squarzoni C, Tillard E, Messad S, Dehecq JS, Gautier A, Martinez D, et al. Tick-borne diseases of cattle in Reunion Island : a sero-epidemiological survey on anaplasmosis, babesiosis and heartwater. Proceedings of the 9th International Symposium on Veterinary Epidemiology and Economics. 2000.

[25] Uilenberg G. Influence du détiquage sur la présence de parasites sanguins chez les bovins malgaches observés après splénectomie. Indications pratiques pour la lutte contre les hématozoaires pathogènes. Rev Elev Med Vet Pays Trop. 1965;18:165–73. 5889950

[26] Keller C, Krüger A, Schwarz NG, Rakotozandrindrainy R, Rakotondrainiarivelo JP, Razafindrabe T, et al. High detection rate of *Rickettsia africae* in *Amblyomma variegatum* but low prevalence of anti-rickettsial antibodies in healthy pregnant women in Madagascar. Ticks Tick Borne Dis. 2016;7:60–5. 10.1016/j.ttbdis.2015.08.005 26318262

[27] Andriamandimby SF, Marianneau P, Rafisandratantsoa J, Rollin PE, Heraud J, Tordo N, et al. Crimean-Congo hemorrhagic fever serosurvey in at-risk professionals, Madagascar, 2008 and 2009. J Clin Virol. 2011;52:370–2. 10.1016/j.jcv.2011.08.008 21889395

[28] Moher D, Liberati A, Tetzlaff J, Altman DG, Altman D, Antes G, et al. Preferred reporting items for systematic reviews and meta-analyses: the PRISMA statement. PLoS Med. 2009;339:1–8. PMC3090117 21603045

[29] Page MJ, McKenzie JE, Bossuyt PM, Boutron I, Hoffmann C, Mulrow CD, et al. The PRISMA 2020 statement: an updated guideline for reporting systematic reviews. BMJ. 2021;372:1–9. 10.1136/bmj.n71 PMC8005924 33782057

[30] Guglielmone A, Nava S, Robbins RG. Geographic distribution of the hard ticks (Acari: Ixodida: Ixodidae) of the world by countries and territories. Zootaxa. 2023;5251:1–274. 10.11646/zootaxa.5251.1.1 37044740

[31] Dantas-Torres F, Mans BJ. Ticks (Ixodida: Argasidae, Ixodidae, Khimairidae, and Nuttalliellidae) of the world. Parasit Vectors. 2026;19:1–17. 10.1186/s13071-026-07493-z PMC13289532 42289755

[32] Uilenberg G, Hoogstraal H, Klein JM. Les Tiques (Ixodea) de Madagascar et leur rôle vecteur. Arch Inst Pasteur Madagascar; Numéro spécial 1979;1–154.

[33] Dupraz M, Toty C, Noël V, Estrada-Peňa A, González-Solís J, Boulinier T, et al. Linking morphometric and genetic divergence with host use in the tick complex, *Ornithodoros capensis* sensu lato. Infect Genet Evol. 2016;46:12–22. 10.1016/j.meegid.2016.10.005 27729267

[34] Dietrich M, Lebarbenchon C, Jaeger A, Le Rouzic C, Bastien M, Lagadec E, et al. *Rickettsia* spp. in seabird ticks from Western Indian Ocean Islands, 2011–2012. Emerg Infect Dis. 2011;20:838–42. 10.3201/eid2005.131088 PMC4012800 24751287

[35] McCoy KD, Dietrich M, Jaeger A, Wilkinson DA, Bastien M, Lagadec E, et al. The role of seabirds of the Iles Eparses as reservoirs and disseminators of parasites and pathogens. Acta Oecol. 2016;72:98–109. 10.1016/j.actao.2015.12.013 PMC7128210 32288503

[36] Converse J, Hoogstraal H, Moussa MI, Feare CJ, Kaiser MN. Soldado Virus (Hughes Group) from *Ornithodoros* (*Alectorobius*) *capensis* (Ixodoidea: Argasidae) infesting sooty tern colonies in the Seychelles, Indian Ocean. Am J Trop Med Hyg. 1975;24:1010–8. 10.4269/ajtmh.1975.24.1010 1200252

[37] Reeves WK, Loftis AD, Sanders F, Spinks MD, Wills W, Denison AM, et al. *Borrelia, Coxiella,* and *Rickettsia* in *Carios capensis* (Acari: Argasidae) from a brown pelican (*Pelecanus occidentalis*) rookery in South Carolina, USA. Exp Appl Acarol. 2006;39:321–9. 10.1007/s10493-006-9012-7 16821092

[38] Wilkinson DA, Dietrich M, Lebarbenchon C, Jaeger A, Le Rouzic C, Bastien M, et al. Massive infection of seabird ticks with bacterial species related to *Coxiella burnetii*. Appl Environ Microbiol. 2014;80:3327–33. 10.1128/AEM.00477-14 PMC4018846 24657860

[39] Hoogstraal H. Description of *Ornithodoros* (*Reticulinasus*) *madagascariensis* n. sp. (lxododidae, Argasidae). Acarologia. 1962;4:186–9.

[40] Soneshine DE, Clifford CM, Kohls GM. The systematics of the subfamily Ornithodorinae (Acarina Argasidae). III. Identification of the larvae of the eastern hemisphere. Ann Entomol Soc Am. 1966;59:92–122. 10.1093/aesa/59.1.92 5966138

[41] Nicholson WL, Sonenshine DE, Noden BH, Brown RN. Chapter 27 - ticks (ixodida). In: Med vet entomol. 2018. p. 603–72.

[42] Roger F, Ratovonjato J, Vola P, Uilenberg G. *Ornithodoros porcinus* ticks, bushpigs, and African swine fever in Madagascar. Exp Appl Acarol. 2001;25:263–9. 10.1023/a:1010687502145 11523921

[43] Uilenberg G. Existence de *Ornithodorus porcinus* Walton, 1962 (Argasidae) à Madagascar. Rev Elev Med Vet Pays Trop. 1963;16:147–50.

[44] Ravaomanana J, Michaud V, Jori F, Andriatsimahavandy A, Roger F, Albina E, et al. First detection of African swine fever virus in *Ornithodoros porcinus* in Madagascar and new insights into tick distribution and taxonomy. Parasit Vectors. 2010;3:1–9. 10.1186/1756-3305-3-115 PMC3012664 21118485

[45] Ševčík M, Špitalská E, Maliterná M, Kabát P, Benda P. First records of *Secretargas transgariepinus* (Argasidae) in Libya and Jordan: corrections of collection records and detection of microorganisms. Parasitol Res. 2024;123:1–10. 10.1007/s00436-024-08239-5 PMC11133098 38805058

[46] Ehlers J, Poppert S, Ratovonamana RY, Ulrich J, Tappe D, Krüger A. Ectoparasites of endemic and domestic animals in southwest Madagascar. Acta Trop. 2019;196:83–92. 10.1016/j.actatropica.2019.05.008 31082365

[47] Klompen H, Apanaskevich DA, Goodman SM. Ixodida, ticks, kongona. In: The new natural history of Madagascar. Princeton University Press; 2022. p. 894–9.

[48] Ehlers J, Krüger A, Jacques S, Yedidya R, Poppert S, Ulrich J, et al. Molecular detection of *Rickettsia *spp., *Borrelia *spp., *Bartonella *spp. and *Yersinia pestis* in ectoparasites of endemic and domestic animals in southwest Madagascar. Acta Trop. 2020;205:1–10. 10.1016/j.actatropica.2020.105339 31935354

[49] Ehlers J, Ganzhorn JU, Silaghi C, Krüger A, Pothmann D, Ratovonamana RY, et al. Tick (*Amblyomma chabaudi*) infestation of endemic tortoises in southwest Madagascar and investigation of tick-borne pathogens. Ticks Tick Borne Dis. 2016;7:378–83. 10.1016/j.ttbdis.2015.12.011 26724898

[50] Durden L, Keirans JE, Smith LL. *Amblyomma geochelone*, a new species of tick (Acari : Ixodidae) from the Madagascan ploughshare tortoise. J Med Entomol. 2002;39:398–403. 10.1603/0022-2585-39.2.398 11931042

[51] Uilenberg G. *Amblyomma loculosum* (Ixodidae): first record from continental Africa. J Med Entomol. 1977;13:516. 10.1093/jmedent/13.4-5.516 845892

[52] Hoogstraal H, Wassef HY, Converse JD, Keirans JE, Clifford CM, Feare CJ. *Amblyomma loculosum* (Ixodoidea: Ixodidae): identity, marine bird and human hosts, virus infection, and distribution in the southern oceans. Ann Entomol Soc Am. 1976;69:3–14.

[53] Perez C, Fontenille D. *Amblyomma loculosum*, sous-genre *Adenopleura*: première découverte à Madagascar. Arch Inst Pasteur Madagascar. 1984;25:145–6. 6534289

[54] Feare CJ, Gill EL. The life cycle of the tick *Ambyomma loculosum* in a sooty tern *Sterna fuscata* colony in the Seychelles. J Zool. 1997;241:643–8.

[55] Walker JB, Olwage A. The tick vectors of *Cowdria ruminantium* (Ixodoidea, Ixodidae, genus *Amblyomma*) and their distribution. Onderstepoort J Vet Res. 1987;54:353–79. 3329325

[56] Barré N, Morel PC. Tiques (Acariens, Ixodoidea) des Mascareignes (Océan Indien) et maladies transmises. Rev Elev Med Vet Pays Trop. 1983;36:371–7. 6675074

[57] Stachurski F, Tortosa P, Rahajarison P, Jacquet S, Yssouf A, Huber K. New data regarding distribution of cattle ticks in the south-western Indian Ocean islands. Vet Res. 2013;44:1–8. 10.1186/1297-9716-44-79 PMC3848863 24016261

[58] Yssouf A, Lagadec E, Bakari A, Foray C, Stachurski F, Cardinale E, et al. Colonization of Grande Comore Island by a lineage of *Rhipicephalus appendiculatus* ticks. Parasit Vectors. 2011;4:1–8. 10.1186/1756-3305-4-38 PMC3069959 21414194

[59] Boucher F, Moutroifi Y, Peba B, Ali M, Moindjie Y, Ruget AS, et al. Tick-borne diseases in the Union of the Comoros are a hindrance to livestock development: circulation and associated risk factors. Ticks Tick Borne Dis. 2020;11:1–9. 10.1016/j.ttbdis.2019.101283. 31519420

[60] Purnell RE. Control of heartwater in cattle in Southern Africa using terramycin/LA. Prev Vet Med. 1984;2:239–54.

[61] Guinat C. La tique dure *Amblyomma variegatum* (Acari, Ixodidae) à Madagascar : détermination de seuils de température pour la métamorphose de la nymphe gorgée à l’adulte. 2012.

[62] Rahajarison P, Arimanana AH, Raliniaina M, Stachurski F. Survival and moulting of *Amblyomma variegatum* nymphs under cold conditions of the Malagasy highlands. Infect Genet Evol. 2014;28:666–75. 10.1016/j.meegid.2014.06.022 24999236

[63] La UG. tique *Amblyomma variegatum* (Ixodidae) apparemment résistante à l’arsenic, à Madagascar. Rev Elev Med Vet Pays Trop. 1975;28:405–8. 1230882

[64] Yssouf A, Socolovschi C, Kernif T, Temmam S, Lagadec E, Tortosa P, et al. First molecular detection of *Rickettsia africae* in ticks from the Union of the Comoros. Parasit Vectors. 2014;7:1–6. 10.1186/1756-3305-7-444 PMC4289259 25245895

[65] Parola P, Barre N. *Rickettsia africae*, agent de la fièvre à tique africaine: un pathogène émergent dans les Antilles et l’Ile de la Réunion. Bull Soc Pathol Exot. 2004;97:193–8. 15462202

[66] Boone I, Henning K, Hilbert A, Neubauer H, von Kalckreuth V, Dekker DM, et al. Are brucellosis, Q fever and melioidosis potential causes of febrile illness in Madagascar? Acta Trop. 2017;172:255–62. 10.1016/j.actatropica.2017.05.013 28502643

[67] Hagen RM, Frickmann H, Ehlers J, Krüger A, Margos G, Hizo-Teufel C, et al. Presence of *Borrelia* spp. DNA in ticks, but absence of *Borrelia* spp. and of *Leptospira* spp. DNA in blood of fever patients in Madagascar. Acta Trop. 2018;177:127–34. 10.1016/j.actatropica.2017.10.002 28986249

[68] Hoogstraal H, Uilenberg G, Klein J-M. *Haemaphysalis *(*Rhipistoma*) *anoplos *sp. n., a spurless tick of the Elongata group (Ixodea, Ixodidae) parasitizing Nesomys rufus Peters (Rodentia) in Madagascar. J Parasitol. 1967;53:1103–5. 6062064

[69] Hoogstraal H, Wassef HY, Uilenberg G. *Haemaphysalis* (*Elongiphysalis*) *elongata* Neumann subgen. n. (Ixodoidea: Ixodidae): structural variation, hosts, and distribution in Madagascar. J Parasitol. 1974;60:480–98. 4833858

[70] Rakotomanga MN, Goodman SM, Soarimalala V, Boyer S, Apanaskevich D. Les tiques dures (Acari: Ixodidae) ectoparasites de micromammifères non-volants dans la forêt d’Ambohitantely. Madagascar Malagasy Nat. 2017;12:59–67.

[71] Hoogstraal H, Camicas JL. *Haemaphysalis *(*Rhipistoma*) *eupleres *(Ixodoidea: Ixodidae), a parasite of the Madagascar falanouc (mongoose): new data and male identity. J Parasitol. 1977;63:1099–109. 10.2307/3279854. 758015

[72] Randimby F, Duplantier JM, Ratovonjato J, Goodman SM, Ramilijaona O, Duchemin J. Analyse de la spécificité parasitaire des tiques Intérêt de la situation de Madagascar et des échantillonnages approfondis. Arch Instit Pasteur Madagascar. 2001;67:49–52. 12471749

[73] Apanaskevich DA, Goodman SM. Description of a new species of *Haemaphysalis* Koch, 1844 (Acari: Ixodidae) from the *H.* (*Rhipistoma*) *asiatica* subgroup, parasite of an endemic Malagasy carnivoran (Carnivora: Eupleridae). Syst Parasitol. 1844;97:591–9. 10.1007/s11230-020-09943-3 33063166

[74] Rodriguez IA, Rasoazanabary E, Godfrey LR. Seasonal variation in the abundance and distribution of ticks that parasitize *Microcebus griseorufus* at the Bezà Mahafaly Special Reserve. Madagascar Int J Parasitol Parasites Wildl. 2015;4:408–13. 10.1016/j.ijppaw.2015.10.007 PMC4683571 26767168

[75] Durden L, Kessler SE, Radespiel U, Hasiniaina AF, Stekolnikov AA, Chalkowski K, et al. Host associations of ectoparasites of the gray mouse lemur, *Microcebus murinus*, in Northwestern Madagascar. J Parasitol. 2021;107:108–14. 10.1645/20-145 33567091

[76] Durden L, Zohdy S, Laakkonen J. Lice and ticks of the eastern rufous mouse lemur, *Microcebus rufus*, with descriptions of the male and third instar nymph of *Lemurpediculus verruculosus* (Phthiraptera: Anoplura). J Parasitol. 2010;96:874–8. 10.1645/GE-2512.1 20950093

[77] Koyama N, Aimi M, Kawamoto Y, Hirai H, Go Y, Ichino S, et al. Body mass of wild ring-tailed lemurs in Berenty reserve, Madagascar, with reference to tick infestation: a preliminary analysis. Primates. 2008;49:9–15. 10.1007/s10329-007-0051-4 17632687

[78] Junge RE, Louis EE. Preliminary biomedical evaluation of wild ruffed lemurs (*Varecia variegata* and *V. rubra*). Am J Primatol. 2005;66:85–94. 10.1002/ajp.20129 15898067

[79] Hokan M, Strube C, Radespiel U, Zimmermann E. Sleeping site ecology, but not sex, affect ecto- and hemoparasite risk, in sympatric, arboreal primates (*Avahi occidentalis* and *Lepilemur edwardsi*). Front Zool. 2017;14:1–12. 10.1186/s12983-017-0228-7 PMC5607495 28943886

[80] Hoogstraal H, Theiler G. Ticks (Ixodoidea, Ixodidae) parasitizing lower primates in Africa, Zanzibar, and Madagascar. J Parasitol. 1959;45:217–22. 13642177

[81] Lado P, Qurollo B, Williams C, Junge R, Klompen H. The microbiome of *Haemaphysalis lemuris* (Acari: Ixodidae), a possible vector of pathogens of endangered lemur species in Madagascar. Ticks Tick Borne Dis. 2018;9:1252–60. 10.1016/j.ttbdis.2018.05.003 29754986

[82] Klein A, Zimmermann E, Radespiel U, Schaarschmidt F, Springer A, Strube C. Ectoparasite communities of small-bodied Malagasy primates: seasonal and socioecological influences on tick, mite and lice infestation of Microcebus murinus and M. ravelobensis in northwestern Madagascar. Parasit Vectors. 2018;11:459. 10.1186/s13071-018-3034-y PMC6083607 30089527

[83] Kiene F, Andriatsitohaina B, Ramsay MS, Rakotondramanana H, Rakotondravony R, Radespiel U, et al. Forest edges affect ectoparasite infestation patterns of small mammalian hosts in fragmented forests in Madagascar. Int J Parasitol. 2020;50:299–313. 10.1016/j.ijpara.2020.01.008 32224123

[84] Uilenberg G, Clifford CM, Hoogstraal H. *Ixodes albignaci* Uilenberg and Hoogstraal: description of male, nymph, and larva, and distribution and hosts in Madagascar (Acarina: Ixodidae). J Med Entomol. 1970;7:611–4. 10.1093/jmedent/7.5.611 5501225

[85] Englert MC, Goodman SM, Apanaskevich DA. Description of a new species of *Ixodes* Latreille, 1795 (Acari: Ixodidae), parasite of shrew tenrecs (Afrotheria: Tenrecidae) and rodents (Rodentia: Muridae) on Madagascar. Syst Parasitol. 2023;100:745–50. 10.1007/s11230-023-10122-3 37874423

[86] Rasoamalala F, Parany MNJ, Rahajandraibe S, Rakotomanga MN, Ramihangihajason T, Soarimalala V, et al. High rickettsial diversity in rodents and their ectoparasites from the Central Highlands of Madagascar. J Med Entomol. 2022;59:667–74. 10.1093/jme/tjab207 34958102

[87] Morel PC. Description of Ixodes (Scaphixodes) domergui n. sp. from Madagascar (Acarina, Ixodoidea). Acarologia. 1966;8:200–7. 5931511

[88] Arthur DR. The *Ixodes schillingsi *group: ticks of Africa and Madagascar, parasitic on primates, with descriptions of two new species (Ixodoidea, Ixodidae). Parasitology. 1957;47:544–59. 10.1017/s0031182000022174 13504868

[89] Blanco MB, Elfawal MA, Durden LA, Beati L, Xu G, Laurie R, et al. Genetic diversity of Ixodid ticks parasitizing eastern mouse and dwarf lemurs in Madagascar, with descriptions of the larva, nymph, and male of *Ixodes lemuris* (Acari : Ixodidae). J Parasitol. 2013;99:11–8. 10.1645/GE-3183.1 22924921

[90] Qurollo BA, Larsen PA, Rakotondrainibe HH, Mahefarisoa K, Rajaonarivelo T, Razafindramanana J, et al. Molecular surveillance of novel tick-borne organisms in Madagascar’s lemurs. Ticks Tick Borne Dis. 2018;9:672–7. 10.1016/j.ttbdis.2018.02.012 29477959

[91] Apanaskevich DA, Soarimalala V, Goodman SM. A new *Ixodes* species (Acari: Ixodidae), parasite of shrew tenrecs (Afrosoricida: Tenrecidae) in Madagascar. J Parasitol. 2013;99:970–2. 10.1645/13-306.1 PMC4833386 23901784

[92] Apanaskevich DA, Goodman SM. Description of three new species of *Ixodes* Latreille, 1795 (Acari: Ixodidae), parasites of tenrecs (Afrotheria: Tenrecidae) on Madagascar. Syst Parasitol. 2020;97:623–37. 10.1007/s11230-020-09944-2 33150511

[93] Olds CL, Mason KL, Scoles GA. *Rhipicephalus appendiculatus* ticks transmit *Theileria parva* from persistently infected cattle in the absence of detectable parasitemia: implications for East Coast fever epidemiology. Parasit Vectors. 2018;11:1-11. 10.1186/s13071-018-2727-6. PMC5834894 29499743

[94] Pienaar R, Latif AA, Mans BJ. Investigations into the host specificity of *Theileria taurotragi*. Vet Parasitol. 2018;254:30–5. 10.1016/j.vetpar.2018.03.003 29657008

[95] Rechav Y, Knight MM, Norval RAI. Life cycle of the Tick *Rhipicephalus evertsi evertsi* Neumann (Acarina: Ixodidae) under Laboratory Conditions. J Parasitol. 1977;63:575–9. 864575

[96] Walker J, Keirans J, Horak I. The genus *Rhipicephalus* (Acari, Ixodidae): a guide to the brown ticks of the world. Cambridge University Press; 2000.

[97] Faye O, Fontenille D, Thonnon J, Gonzalez J-P, Cornet JP, Camicas J-L. Transmission expérimentale du virus de la fièvre hémorragique de Crimée-Congo par la tique *Rhipicephalus evertsi evertsi* (Acarina : Ixodidae). Virologie. 1999;92:143–8. 10472436

[98] Kamani J, Sacristán Yagüe I, Shand M, Okosi IR, Olubade T, Moses SA, et al. Genetic characterization of *Rhipicephalus microplus* (Acari: Ixodidae) in Africa reveals the presence of diverse haplotypes within a single clade: implications for pathogens transmission and tick control in a predominantly pastoral production system. Acta Trop. 2025;271:1–9. 10.1016/j.actatropica.2025.107869 41093088

[99] Futse JE, Ueti MW, Knowles DP, Palmer GH. Transmission of *Anaplasma marginale* by *Boophilus microplus*: retention of vector competence in the absence of vector-pathogen interaction. J Clin Microbiol. 2003;41:3829–34. 10.1128/JCM.41.8.3829-3834.2003 PMC179812 12904396

[100] Obregón D, Corona González B, Díaz Sánchez AA, Armas Y, Roque E, de OliveiraS MC, et al. Efficient transovarial transmission of *Babesia *spp in *Rhipicephalus microplus *ticks fed on water buffalo (*Bubalus bubalis*). Pathogens. 2020;9:1–10. 10.3390/pathogens9040280 PMC7238136 32290468

[101] Uilenberg G. Observations complémentaires faites à Madagascar sur la résistance de la tique *Boophilus microplus* (Canestrini) aux insecticides de la famille des hydrocarbures chlorés. Rev Elev Med Vet Pays Trop. 1967;20:301–6. 5614032

[102] Dorla E, Grondin I, Hue T, Clerc P, Dumas S, Gauvin-Bialecki A, et al. Traditional uses, antimicrobial and acaricidal activities of 20 plants selected among Reunion Island’s flora. S Afr J Bot. 2019;122:447–56.

[103] Rabenandrasana MAN, Habib A, Tantely ML, Rodrigues V, Harimanana AN, Andriamandimby SF, et al. Molecular detection of *Ehrlichia ruminantium* in ticks from ruminants during the 2021 Rift valley fever outbreak in Mananjary. Madagascar Parasitol Res. 2025;124:1–7. 10.1007/s00436-025-08508-x PMC12373694 40844790

[104] Mathiot CC, Fontenille D, Digoutte J, Coulanges P. First isolation of Congo-crimean haemorrhagic fever virus in Madagascar. Ann Inst Pasteur Virol. 1988;139:239–41. 10.1016/s0769-2617(88)80022-4 3144994

[105] Burt FJ, Swanepoel R. Molecular epidemiology of African and Asian Crimean-Congo haemorrhagic fever isolates. Epidemiol Infect. 2005;133:659–66. 10.1017/s0950268805003730 PMC2870293 16050511

[106] Alkishe A, Cobos ME, Peterson AT, Samy AM. Recognizing sources of uncertainty in disease vector ecological niche models: an example with the tick *Rhipicephalus sanguineus* sensu lato. Perspect Ecol Conserv. 2020;18:91–102.

[107] Lee GKC, Ignace JAE, Robertson ID, Irwin PJ. Canine vector-borne infections in Mauritius. Parasit Vectors. 2015;8:1–7. 10.1186/s13071-015-0790-9 PMC4369807 25889893

[108] Apanaskevich DA, Greiman SE, Goodman SM, Apanaskevich MA, Rokeyaahmed Barker SC. Validation of Sharifiella Santos Dias,1958 (Acari: Ixodidae) as a genus of hard ticks containing S. theilerae (Hoogstraal, 1953), a parasite of tail-less tenrec, Tenrec ecaudatus (Schreber) (Afrosoricida: Tenrecidae) on Madagascar. Zootaxa. 2025;5627:503–25. 10.11646/zootaxa.5627.3.5 41119239

[109] Li Z, Chen W, Qiu Z, Li Y, Fan J, Wu K, et al. African swine fever virus: a review. Life. 2022;12:1–41. 10.3390/life12081255 PMC9409812 36013434

[110] Rousset D, Randriamparany T, Maharavo Rahantamalala CY, Randriamahefa N, Zeller H, Rakoto-Andrianarivelo M, et al. Introduction de la peste porcine africaine à Madagascar, histoire et leçons d’une émergence. Arch Inst Pasteur Madagascar. 2001;67:31–3. 12471744

[111] Jori F, Bastos A, Boinas F, Van HJ, Heath L, Martinez-lopez B, et al. An updated review of *Ornithodoros* ticks as reservoirs of African swine fever in sub-saharan Africa and Madagascar. Pathogens. 2023;12:1–22. 10.3390/pathogens12030469 PMC10059854 36986391

[112] Ravaomanana J, Jori F, Vial L, Pérez-Sánchez R, Blanco E, Michaud V, et al. Assessment of interactions between African swine fever virus, bushpigs (*Potamochoerus larvatus*), *Ornithodoros* ticks and domestic pigs in north-western Madagascar. Transbound Emerg Dis. 2011;58:247–54. 10.1111/j.1865-1682.2011.01207.x 21320295

[113] Sánchez-Vizcaíno JM, Mur L, Martínez-López B. African swine fever: an epidemiological update. Transbound Emerg Dis. 2012;59:27–35. 10.1111/j.1865-1682.2011.01293.x 22225967

[114] Jori F, Vial L, Penrith ML, Pérez-Sánchez R, Etter E, Albina E, et al. Review of the sylvatic cycle of African swine fever in sub-saharan Africa and the Indian ocean. Virus Res. 2013;173:212–27. 10.1016/j.virusres.2012.10.005 23142551

[115] Kocan KM, de la Fuente J, Blouin EF, Coetzee JF, Ewing SA. The natural history of *Anaplasma marginale*. Vet Parasitol. 2010;167:95–107. 10.1016/j.vetpar.2009.09.012 19811876

[116] Aubry P, Geale DW. A review of Bovine anaplasmosis. Transbound Emerg Dis. 2011;58:1–30. 10.1111/j.1865-1682.2010.01173.x 21040509

[117] Atif FA, Mehnaz S, Qamar MF, Roheen T, Sajid MS, Ehtisham-Ul-haque S, et al. Epidemiology, diagnosis, and control of canine infectious cyclic thrombocytopenia and granulocytic anaplasmosis: emerging diseases of veterinary and public health significance. Vet Sci. 2021;8:1–20. 10.3390/vetsci8120312 PMC8705095 34941839

[118] Yao XY, Yu SQ, Tian N, Wang F, Li SZ, Li LH. Nucleic acid prevalence of zoonotic *Babesia* in humans, animals and questing ticks, a systematic review and meta-analysis. Trop Med Infect Dis. 2023;8:1–7. 10.3390/tropicalmed8030132 PMC10051474 36977133

[119] Homer MJ, Aguilar-Delfin I, Telford SR III, Krause PJ, Persing DH. Babesiosis. Clin Microbiol Rev. 2000;3:451–69. 10.1128/cmr.13.3.451-469.2000 PMC88943 10885987

[120] Lobato E, Pearce-Duvet J, Staszewski V, Gómez-Díaz E, González-Solís J, Kitaysky A, et al. Seabirds and the circulation of lyme borreliosis bacteria in the north pacific. Vector Borne Zoonotic Dis. 2011;11:1521–7. 10.1089/vbz.2010.0267 21919724

[121] Gern L. Life cycle of *Borrelia burgdorferi* sensu lato and transmission to humans. Curr Probl Dermatol. 2009;37:18–30. 10.1159/000213068 19367095

[122] Duplantier J-M, Duchernin ]B. Human diseases and introduced small mammals. In: The natural history of Madagascar. The University of Chicago; 2003;159–61.

[123] Rodhain PF, Fontenille D. La récurrente à tiques malgache: une affection éradiquée. Bull Soc Pathol Exot. 1989;82:192–8. 2743524

[124] Dinkisa G. Review on control of cowdriosis in ruminants. J Vet Med Res. 2018;5:1–7.

[125] Yunker CE. Heartwater in sheep and goats: a review. Onderstepoort J Vet Res. 1996;63:159–70. 8856765

[126] Camus E, Fourrier J-C, Vely M. Présence de la cowdriose (heartwater) à Mayotte (Océan Indien). Rev Elev Med Vet Pays Trop. 1998;51:282.

[127] Jori F, Godfroid J, Michel AL, Potts AD, Jaumally MR, Sauzier J, et al. An assessment of zoonotic and production limiting pathogens in rusa deer (*Cervus timorensis rusa*) from Mauritius. Transbound Emerg Dis. 2013;61:31–42. 10.1111/tbed.12206 24382104

[128] Baneth G, Samish M, Alekseev E, Aroch I, Shkap V. Transmission of *Hepatozoon Canis* to dogs by naturally-fed or percutaneously-injected *Rhipicephalus sanguineus* ticks. J Parasitol. 2001;87:606–11. 10.1645/0022-3395(2001)087[0606:TOHCTD]2.0.CO;2 11426725

[129] Daskalaki AA, Ionică AM, Jeetah K, Gherman CM, Mihalca AD. Molecular confirmation of *Hepatozoon canis* in Mauritius. Acta Trop. 2018;177:116–7. 10.1016/j.actatropica.2017.10.005 29024615

[130] Wennerås C. Infections with the tick-borne bacterium *Candidatus Neoehrlichia mikurensis*. Clin Microbiol Infect. 2015;21:621–30. 10.1016/j.cmi.2015.02.030 25770773

[131] Silaghi C, Beck R, Oteo JA, Pfeffer M, Sprong H. Neoehrlichiosis: an emerging tick-borne zoonosis caused by *Candidatus Neoehrlichia mikurensis*. Exp Appl Acarol. 2015;68:279–97. 10.1007/s10493-015-9935-y 26081117

[132] Million M, Raoult D. Recent advances in the study of Q fever epidemiology, diagnosis and management. J Infect. 2015;71:S2-9. 10.1016/j.jinf.2015.04.024 25917809

[133] Celina SS, Cerny J. *Coxiella burnetii* in ticks, livestock, pets and wildlife: a mini-review. Front Vet Sci. 2022;9:1–13. 10.3389/fvets.2022.1068129 PMC9691889 36439350

[134] Jaubert J, Naze F, Camuset G, Larrieu S, Pascalis H, Guernier V, et al. Seroprevalence of *Coxiella burnetii* (Q fever) exposure in humans on Reunion Island. Open Forum Infect Dis. 2019;6:1–4. 10.1093/ofid/ofz227 PMC6602885 31281854

[135] Cardinale E, Esnault O, Beral M, Naze F, Michault A. Emergence of *Coxiella burnetii* in ruminants on Reunion Island? Prevalence and risk factors. PLoS Negl Trop Dis. 2014;8:1–8. 10.1371/journal.pntd.0003055 PMC4125295 25101780

[136] Mahajan SK. Rickettsial Diseases. JAPI. 2012;60:33–44. 23405539

[137] Mayoux A-M. Enquêtes sur les rickettsioses à Madagascar II. *Rickettsia conori*: fièvre par morsure de tiques. Arch Inst Pasteur Madagascar. 1972;41:49–54.

[138] Gérardin P, Zemali N, Bactora M, Camuset G, Balleydier E, Pascalis H, et al. Seroprevalence of typhus group and spotted fever group *Rickettsia* exposures on Reunion Island. BMC Res Notes. 2019;12:1–5. 10.1186/s13104-019-4416-8 PMC6617902 31288833

[139] Abdela N, Bekele T. Bovine theileriosis and its control: a review. Adv Biol Res. 2016;10:200–12.

[140] Kwasnik M, Rola J, Rozek W. Tick-borne encephalitis-review of the current status. J Clin Med. 2023;12:1–13. 10.3390/jcm12206603 PMC10607749 37892741

[141] Dellagi K, Salez N, Maquart M, Larrieu S, Yssouf A, Silaï R, et al. Serological evidence of contrasted exposure to arboviral infections between Islands of the Union of Comoros (Indian Ocean). PLoS Negl Trop Dis. 2016;10:1–14. 10.1371/journal.pntd.0004840 PMC5157944 27977670

[142] Klein J. Description d’une nouvelle tique endémique de Madagascar *Ornithodoros grenieri* sp. n. (Ixodidae, Argasidae), parasite du rongeur cricétidé, Hypogeomys antimena Grandidier, 1969. Bull Soc Pathol Exot. 1964;57:1276–89. 5897648

[143] Morel PC, Vassiliades G. Description de Argas hoogstraali n sp de Madagascar (Acariens, Ixodea). Acarologia. 1965;7:268–73.

[144] Hoogstraal H, Uilenberg G, Blanc CP. The *Argas* subgenus *Secretargas* (Ixodoidea, Argasidae) in Madagascar: descriptions of *A. *(*S.*)* echinops*, new species, and of the adults and nymph of *A. *(*S.*) *hoogstraali*. Ann Entomol Soc Am. 1967;60:149–62.

[145] Mans BJ, de Klerk D, Pienaar R, de Castro MH, Latif AA. The mitochondrial genomes of *Nuttalliella namaqua* (Ixodoidea: Nuttalliellidae) and *Argas africolumbae* (Ixodoidae: Argasidae): estimation of divergence dates for the major tick lineages and reconstruction of ancestral blood-feeding characters. PLoS ONE. 2012;7:1–12. 10.1371/journal.pone.0049461 PMC3493528 23145176

[146] Beati L, Klompen H. Phylogeography of Ticks (Acari: Ixodida). Annu Rev Entomol. 2019;64:379–97. 10.1146/annurev-ento-020117-043027 30354695

[147] Poux C, Madsen O, Marquard E, Vieites DR, De Jong WW, Vences M. Asynchronous colonization of Madagascar by the four endemic clades of primates, tenrecs, carnivores, and rodents as inferred from nuclear genes. Syst Biol. 2005;54:719–30. 10.1080/10635150500234534 16243759

[148] Socolovschi C, Huynh TP, Davoust B, Gomez J, Raoult D, Parola P. Transovarial and trans-stadial transmission of *Rickettsiae africae* in *Amblyomma variegatum* ticks. Clin Microbiol Infect. 2009;15:317–8. 10.1111/j.1469-0691.2008.02278.x 19456811

[149] Camus E, Barré N. The role of *Amblyomma variegatum* in the transmission of heartwater with special reference to Guadeloupe. Ann N Y Acad Sci. 1992;653:33–41. 10.1111/j.1749-6632.1992.tb19627.x 1626882

[150] Dantas-Torres F, de Sousa-Paula LC, Otranto D. The *Rhipicephalus sanguineus* group: updated list of species, geographical distribution, and vector competence. Parasit Vectors BioMed Central Ltd. 2024;17:1–31. 10.1186/s13071-024-06572-3 PMC11681662 39731169

[151] Groves MG, Dennis GL, Amyx HL, Huxsoll DL. Transmission of *Ehrlichia canis* to dogs by ticks (*Rhipicephalus sanguineus*). Am J Vet Res. 1975;36:937–40. 1147359

[152] Howell JM, Ueti MW, Palmer GH, Scoles GA, Knowles DP. Persistently infected calves as reservoirs for acquisition and transovarial transmission of *Babesia bovis* by *Rhipicephalus* (*Boophilus*) *microplus*. J Clin Microbiol. 2007;45:3155–9. 10.1128/JCM.00766-07 PMC2045367 17687016

[153] de la Fournière S, Guillemi EC, Paoletta MS, Pérez A, Obregón D, Cabezas-Cruz A, et al. Transovarial Transmission of *Anaplasma marginale* in *Rhipicephalus* (*Boophilus*) *microplus* ticks results in a bottleneck for train diversity. Pathogens. 2023;12:1–14. 10.3390/pathogens12081010 PMC10459439 37623970

[154] Krishnavajhala A, Armstrong BA, Lopez JE. Vector competence of geographical populations of *Ornithodoros turicata* for the tick-borne relapsing fever spirochete *Borrelia turicatae*. Appl Environ Microbiol. 2018;84:1–9. 10.1128/AEM.01505-18 PMC6193384 30143510

[155] Messina JP, Pigott DM, Golding N, Duda KA, Brownstein JS, Weiss DJ, et al. The global distribution of Crimean-Congo hemorrhagic fever. Trans R Soc Trop Med Hyg. 2015;109:503–13. 10.1093/trstmh/trv050 PMC4501401 26142451

[156] Nasirian H. New aspects about Crimean-Congo hemorrhagic fever (CCHF) cases and associated fatality trends: a global systematic review and meta-analysis. Comp Immunol Microbiol Infect Dis. 2020;69:1–56. 10.1016/j.cimid.2020.101429 32062190

[157] Celina SS, Černý J, Samy AM. Mapping the potential distribution of the principal vector of Crimean-Congo haemorrhagic fever virus *Hyalomma marginatum* in the Old World. PLoS Negl Trop Dis. 2023;17:1–19. 10.1371/journal.pntd.0010855 PMC10703407 38011221

[158] Foucher A, Evrard O, Rabiet L, Cerdan O, Landemaine V, Bizeul R, et al. Uncontrolled deforestation and population growth threaten a tropical island’s water and land resources in only 10 years. Sci Adv. 2024;10:1–19. 10.1126/sciadv.adn5941 PMC11323937 39141744

[159] Andrianambinina FOD, Ganzhorn JU, Waeber PO, Wilmé L. Complex deforestation patterns in and around the protected areas of Madagascar from 2015 to 2023. Land. 2025;14:1–18.

[160] Koenig XGH, Deenapanray PNK. Land use and environmental degradation in the island state of Mauritius: governance and problem conceptions. Land Use Policy. 2024;146:1–16.

[161] Gottwalt A. Impacts of deforestation on vector-borne disease incidence deforestation, an ongoing problem in the developing world, creates ideal conditions for vectors to breed and spread infectious diseases. Columbia Univ J Glob Health. 2014;3:16–9.

[162] Magnier J, Druet T, Naves M, Ouvrard M, Raoul S, Janelle J, et al. The genetic history of Mayotte and Madagascar cattle breeds mirrors the complex pattern of human exchanges in Western Indian Ocean. Genes Genomes Genet. 2022;12:1–14. 10.1093/g3journal/jkac029 PMC8982424 35137043

[163] Robequain C. Destin d’une île à sucre : l’économie et le peuplement de Maurice. Ann Geogr. 1954;63:255–73.

[164] Goodman SM, Olson LE, Nicoll ME. Tenrecidae: *Tenrec ecaudatus*, tailless tenrec, tandraka, trandraka, kelora. In: Goodman SM, editor. The new natural history of Madagascar. Princeton: Princeton University Press; 2022. p. 1891–4.

[165] Walsh MT. Island subsistence: hunting, trapping and the translocation of wildlife in the Western Indian Ocean. Azania. 2007;42:83–113.

[166] Balboa RF, Bertola LD, Brüniche-Olsen A, Rasmussen MS, Liu X, Besnard G, et al. African bushpigs exhibit porous species boundaries and appeared in Madagascar concurrently with human arrival. Nat Commun. 2024;15:1–15. 10.1038/s41467-023-44105-1 PMC10764920 38172616

[167] Boivin N, Crowther A, Helm R, Fuller DQ. East Africa and Madagascar in the Indian Ocean world. J World Prehist. 2013;26:213–81.

[168] Barré N, Uilenberg G. Propagation de parasites transportés avec leurs hôtes: cas exemplaires de deux espèces de tiques du bétail. Revue scientifique et technique-Office international des épizooties. 2010;29:135–47.

[169] Charmoy DD. Summary of investigation on insect pests during the five months. Mauritius Department of Agriculture, Division of Entomology. 1915.

[170] Gillard A. Étude des tiques et de la babesiellose bovine à la Réunion. Rev. Agric. Ile de la Réunion. 1949.

[171] Kariuki DP, Young AS, Morzaria SP, Lesan IAC, Mining SK, Omwoyo P, et al. *Theileria parva* carrier state in naturally infected and artificially immunised cattle. Trop Anim Health Prod. 1995;27:15–25. 10.1007/BF02236328 7770947

[172] Carroll SA, Reynes J-M, Khristova ML, Andriamandimby SF, Rollin PE, Nichol ST. Genetic evidence for Rift valley fever outbreaks in Madagascar resulting from virus introductions from the east African mainland rather than enzootic maintenance. J Virol. 2011;85:6162–7. 10.1128/JVI.00335-11 PMC3126502 21507967

[173] Kim Y, Métras R, Dommergues L, Youssouffi C, Combo S, Le Godais G, et al. The role of livestock movements in the spread of rift valley fever virus in animals and humans in Mayotte, 2018–19. PLoS Negl Trop Dis. 2021;15:1–19. 10.1371/journal.pntd.0009202 PMC7939299 33684126

[174] Lancelot R, Béral M, Rakotoharinome VM, Andriamandimby SF, Héraud JM, Coste C, et al. Drivers of Rift valley fever epidemics in Madagascar. Proc Natl Acad Sci. 2017;114:938–43. 10.1073/pnas.1607948114 PMC5293023 28096420

[175] Allibert C. Le site de Dembeni (Mayotte, Archipel des Comores): Mission 1984. Etudes Océan Indien. 1989.

[176] Allibert C, Vérin P. The early pre-Islamic history of the Comoros Islands: links with Madagascar and Africa. In: The Indian Ocean in antiquity. London: Kegan Paul International; 1996. p. 461–70.

[177] Cheke A, Hume JP. Lost land of the dodo: an ecological history of Mauritius, Réunion and Rodrigues. London: T & A D Poyser; 2008.

[178] Cheke A. The timing of arrival of humans and their commensal animals on Western Indian Ocean oceanic islands. Phelsuma. 2010;18:38–69.

[179] Racey PA, Nicoll ME. Mammals of the Seychelles. In: The biogeography and ecology of the Seychelles islands. The Hague: Junk; 1984. p. 607–26.

[180] Gomard Y, Goodman SM, Soarimalala V, Turpin M, Lenclume G, Ah-Vane M, et al. Co-radiation of *Leptospira* and Tenrecidae (Afrotheria) on Madagascar. Trop Med Infect Dis. 2022;7:1–8. 10.3390/tropicalmed7080193 PMC9415048 36006285

[181] Lagadec E, Gomard Y, Le Minter G, Cordonin C, Cardinale E, Ramasindrazana B, et al. Identification of *Tenrec ecaudatus*, a wild mammal introduced to Mayotte Island, as a reservoir of the newly identified human pathogenic *Leptospira mayottensis*. PLoS Negl Trop Dis. 2016;10:1–12. 10.1371/journal.pntd.0004933 PMC5004980 27574792

[182] Andoh M, Sakata A, Takano A, Kawabata H, Fujita H, Une Y, et al. Detection of *Rickettsia* and *Ehrlichia* spp. in ticks associated with exotic reptiles and amphibians imported into Japan. PLoS ONE. 2015;10:1–14. 10.1371/journal.pone.0133700 PMC4514593 26207382

[183] Sparagano O, George D, Giangaspero A, Špitalská E. Arthropods and associated arthropod-borne diseases transmitted by migrating birds. The case of ticks and tick-borne pathogens. Vet Parasitol. 2015;213:61–6. 10.1016/j.vetpar.2015.08.028 26343302

[184] Bellard C, Rysman JF, Leroy B, Claud C, Mace GM. A global picture of biological invasion threat on islands. Nat Ecol Evol. 2017;1:1862–9. 10.1038/s41559-017-0365-6 29109470

